# Complete disruption of gD binding to HVEM alters virus replication and host receptor homeostasis

**DOI:** 10.1371/journal.ppat.1014566

**Published:** 2026-09-08

**Authors:** Deepak Arya, Kati Tormanen, Jay J. Oh, Shaohui Wang, Homayon Ghiasi

**Affiliations:** Center for Neurobiology and Vaccine Development, Ophthalmology Research, Department of Surgery, Cedars-Sinai Health Sciences University, Cedars-Sinai Medical Center, Los Angeles, California, United States of America; Oklahoma State University College of Osteopathic Medicine, UNITED STATES OF AMERICA

## Abstract

Herpes simplex virus 1 (HSV‑1) establishes infection through coordinated interactions of multiple viral glycoproteins with host cell receptors. Interactions between the viral glycoprotein D (gD) and the immunomodulatory cellular receptor HVEM (herpesvirus entry mediator) critically influence HSV‑1 latency and reactivation by regulating both receptor engagement and immunomodulatory signaling in the corneal epithelium, immune cells, and neurons. The underlying mechanisms of these phenotypes remain poorly understood because no tools selectively block their interaction. To dissect the contributions of individual amino acids (aa) to HVEM binding, we co‑immunoprecipitated wild‑type (WT) and mutant (Q27P and Q27A‑L28A‑T29A) forms of gD that disrupt gD binding to HVEM. We found that gD aa Q27-T29 were critical for HVEM binding, with the triple mutant showing greater disruption of HVEM binding than the Q27P single mutation, as assessed by co-immunoprecipitation and immunostaining. To map gD binding to HVEM and assess functional relevance, we constructed two recombinant viruses: a single-amino-acid (aa) gD mutation (v27 virus) and a triple-aa mutation in gD (v27-29 virus). Replication of both mutant viruses was comparable to that of the WT virus in four mammalian cell lines, except in Neuro2a cells. However, using confocal microscopy, HVEM protein expression was reduced in v27-29-infected Neuro2a cells, with no gD colocalization observed. Although Neuro2a cells infected with the single gD v27 mutant also showed less HVEM-gD colocalization than Neuro2a cells infected with WT virus, gD protein levels were not affected. RT‑PCR analysis of Neuro2a cells infected with single and triple gD mutants showed significantly less expression of viral glycoproteins (gB, gC, gD), host receptors (HVEM, nectin‑1, 3‑O‑sulfated heparan sulfate), and HVEM ligands (BTLA, LIGHT, Ltα, CD160), with the most pronounced effects observed for the triple v27‑29 mutant virus. Together, these results show that aa Q27-T29, rather than aa Q27 alone, are essential to completely block gD binding to HVEM and, consequently, affect overall receptor homeostasis.

## Author summary

Previously, we demonstrated that loss of HVEM impairs HSV-1 reactivation without significantly affecting primary viral replication. As a member of the TNF receptor superfamily, HVEM functions as a key immunomodulatory receptor and a critical entry mediator for HSV-1 through its interaction with glycoprotein D (gD). Beyond facilitating viral entry, gD–HVEM engagement can influence host immune signaling pathways, thereby shaping the local immune environment during infection. In this study, we identify amino acids 27–29 of gD as essential determinants for HVEM binding. These findings provide mechanistic insight into how HSV-1 exploits specific receptor interactions not only to gain entry but also to modulate immune responses. Disruption of the gD–HVEM interface, therefore, represents a promising strategy to limit viral spread and reactivation while potentially preserving antigen presentation and immune recognition.

## Introduction

HSV‑1 is a highly prevalent human pathogen that establishes lifelong infection and recurrent disease [1,2]. Ocular HSV infection can progress to herpes stromal keratitis (HSK), a chronic immune‑inflammatory corneal disease that is a significant cause of infectious blindness [3–5]. Primary HSV infection is initiated through coordinated interactions between viral glycoproteins and host entry receptors on epithelial or mucosal cells that mediate attachment, entry, and immune responses [6–8], including those of the corneal surface [4,9,10]. Viral spread to the trigeminal ganglia (TG) then occurs, where latency is established in sensory neurons and sporadic reactivation drives repeated corneal inflammation and tissue damage [2,11,12]. Despite extensive knowledge of HSV infection processes, the immunological consequences of HVEM-gD engagement in viral infection remain poorly defined.

An important HSV glycoprotein, gD, plays a key role in virus infection and pathogenesis as a major inducer and target of humoral and cell-mediated immune responses [13–21]. Cellular receptors for gD include nectin-1 [22,23], HVEM [24–26], and 3-OS-HS [23]. gD is the primary viral protein that engages the HVEM receptor during primary HSV infection [26]. The contact between gD amino acids (aa) 27–29 and HVEM aa 35–37 produces a β‑sheet interface stabilized by hydrogen bonds, van der Waals interactions, and electrostatic forces. This gD-HVEM complex mimics the interaction between HVEM and the B and T lymphocyte attenuator (BTLA) [27], thereby allowing the virus to directly access NF-κB-dependent cell survival pathways through HVEM. We previously showed that LAT upregulates HVEM via sncRNA1 and sncRNA2, while other gD receptors, such as nectin-1 and 3-OS-HS, remained unchanged during latency, suggesting a significant role for HVEM in latency establishment and reactivation [28,29].

HVEM is distinct in that it serves as both an entry receptor and a regulator of the host immune response. As a member of the TNF receptor superfamily, HVEM orchestrates a signaling balance between stimulatory and inhibitory pathways by engaging ligands such as LIGHT (TNFSF14), lymphotoxin-α (LTα), BTLA, and CD160 [30–34]. HVEM is selectively upregulated during latency, and both HSV latency and reactivation are significantly attenuated in *HVEM*^*-/-*^ mice compared with WT control mice [28]. Deletion of BTLA, LIGHT, LTα, or CD160 impairs efficient establishment of latency without significantly altering reactivation [35,36], suggesting that HSV-1 specifically exploits HVEM itself rather than its standard signaling partners to promote reactivation. Such specificity indicates a structural basis for HSV-1 gD stabilization or for HVEM dysregulation independent of endogenous ligands. Thus, LAT-dependent upregulation of HVEM enhances gD's interaction with HVEM and promotes both primary and latent HSV-1 infections.

To determine the structural basis of this interaction, we performed co-IP assays in HEK293T cells expressing mouse HVEM with either WT or mutant gD constructs that block HVEM binding. Mutation of a single gD aa, Q27 (Q27P), reduced HVEM binding, while triple-aa mutations (Q27A-L28A-T29A) completely blocked it. We generated recombinant HSV-1 mutants carrying these substitutions (v27 with Q27P and v27-29 with Q27A-L28A-T29A) in a GFP-McKrae background and verified them by whole genome sequencing. These gD mutant viruses are replicated comparably in several mammalian cell lines but differ in Neuro2a cells. Strikingly, viral glycoproteins and host HVEM receptor expressions were significantly reduced in the mutant viruses, with the triple v27-29 virus showing the most pronounced effect. Together, these results clearly indicate that gD-HVEM interactions extend beyond viral entry to influence receptor stability and expression.

## Results

### Mutation of gD aa 27-–29 completely abolishes binding to HVEM

**Fig 1:**
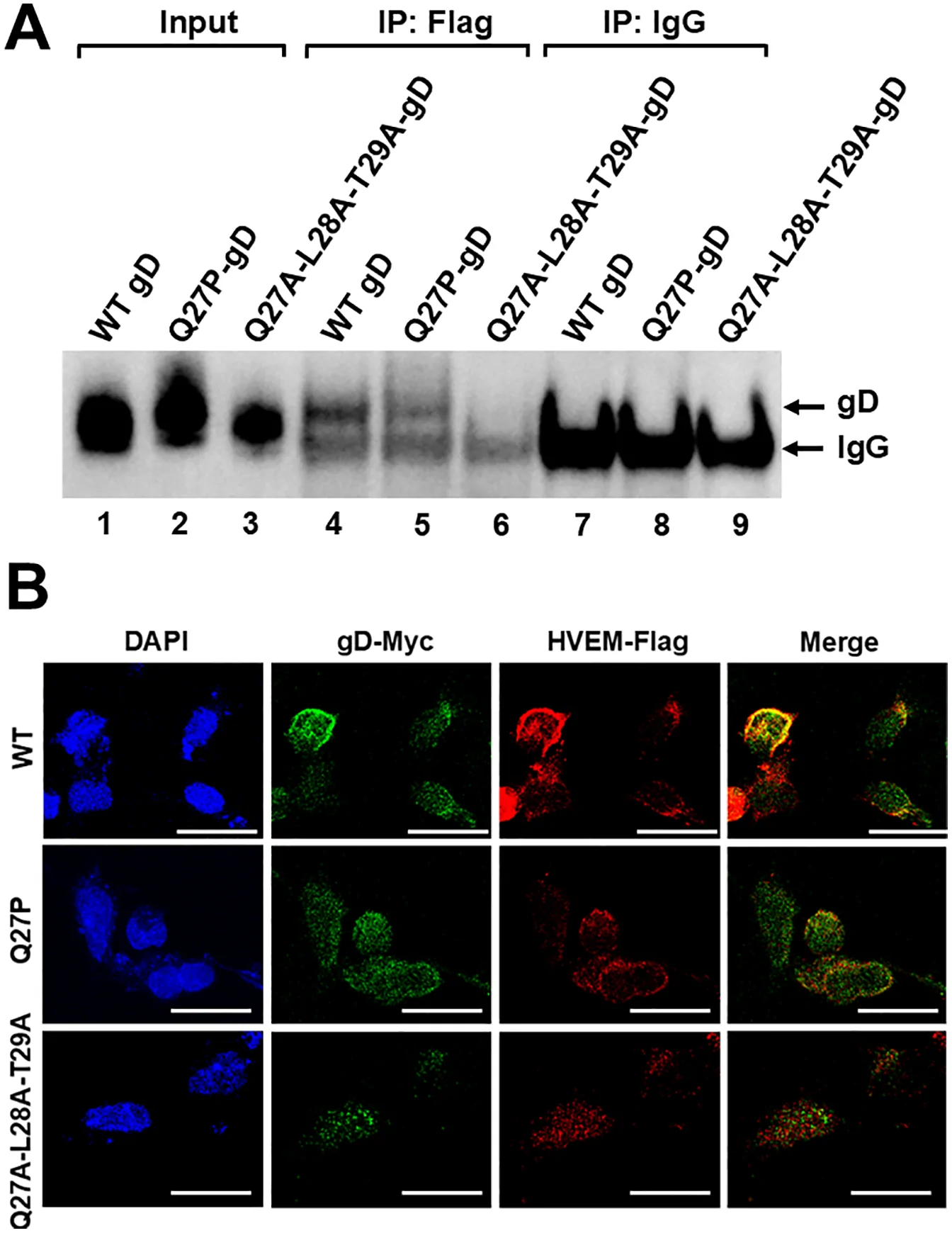
gD mutations at aa 27–29 abolish binding to HVEM *in vitro.* HEK293T cells were transfected with mouse HVEM-2xFlag and either WT-gD, Q27P-gD, or Q27A-L28A-T29A-gD DNA plasmids at a 1:1 ratio for 24 hr. A) Co-immunoprecipitation showing reduced gD binding to HVEM *in vitro*. Cellular lysates were incubated with anti-Flag antibody bound to IgG beads, and the resulting immunoprecipitated was analyzed by western blot with anti-myc antibody; and B) Confocal microscopy showing gD mutations at aa 27–29 (3X) abolish binding to HVEM *in vitro*. HEK293T cells were transfected with plasmids encoding HVEM-2xFlag and either WT-gD, Q27P-gD, or Q27A-L28A-T29A-gD tagged with myc for 72 h. Transfected cells were fixed, immunostained with anti-myc and anti-Flag antibodies, and analyzed by confocal microscopy (see Materials and Methods). Panels show representative transfected HEK293T cells. Scale bar, 25 μm.

Mutating aa glutamine at gD position 27 to a proline has been shown to eliminate gD binding to HVEM [24,37]. This group also reported that gD aa that mediate contact with HVEM is clustered near the intermolecular antiparallel β‑sheet interface that is stabilized by hydrogen bonds between gD aa 27–29 and HVEM aa 35–37. Because a single mutation at aa Q27 can shift receptor usage from HVEM toward nectin‑1, it is reasonable to propose that additional mutations within aa 27–29 could affect binding to both HVEM and nectin‑1, given that each receptor engages overlapping gD surfaces, although the precise contributions to each receptor interface may differ. Based on this information, we generated two transgenic mouse strains in an *HVEM*^*−/−*^ mouse background using mutated forms of mouse and human HVEM [29]. Infection of these mice, along with their controls, suggested that more than one discrete region of gD may contribute to HVEM binding. To map sites on viral gD that are necessary for its interaction with HVEM, we created three gD constructs: a full-length WT-gD open reading frame (ORF) tagged with myc (WT-gD), a single-point mutant with a Q27P substitution (glutamine to proline), and a triple mutant with Q27A-L28A-T29A substitutions. A full-length mouse HVEM ORF with dual Flag tags was also created, and all constructs were cloned into a pcDNA3.1 vector. HEK293T cells were co-transfected with mouse HVEM-2xFlag and either WT-gD, Q27P-gD, or Q27A-L28A-T29A-gD plasmids. Protein interactions were examined by anti-Flag IP followed by anti-gD Western blot. WT-gD showed strong co-immunoprecipitation with HVEM (Fig 1A**, IP: Flag Ab, WT-gD lane 4**). The binding of Q27P-gD to HVEM was less than that of WT-gD, but not completely abolished (Fig 1A**, IP: Flag Ab, Q27P-gD lane 5**), whereas the binding of Q27A-L28A-T29A-gD was undetectable (Fig 1A**, IP: Flag Ab, Q27A-L28A-T29A-gD lane 6**). No gD signal appeared in IgG control IPs from cells transfected with any construct (Fig 1A**, IP: IgG Ab lanes 7–9**). These results suggest that rather than a single aa, HVEM binding requires coordinated interactions across the Q27-L28-T29 motif in the gD N-terminus.

To confirm the pull-down experiments described in Fig 1A, HEK293T cells were transfected with HVEM-2xFlag + WT-gD myc, HVEM-2xFlag + Q27P-gDmyc, or HVEM-2xFlag + Q27A-L28A-T29A-gDmyc for 72 hr as described in Materials and Methods. Transfected cells were fixed and immunostained with anti-myc and anti-Flag antibodies. WT-gD strongly co-localized with HVEM (Fig 1B**, merge WT-gD + HVEM panel**); binding of Q27P-gD to HVEM was less than that of WT-gD (Fig 1B**, merge of Q27P-gD + HVEM panel**), and binding of Q27A-L28A-T29A-gD to HVEM was not detectable compared to either WT or Q27P-gD (Fig 1B**, merge of Q27A-L28A-T29A-gD + HVEM panel**). These imaging data were supported by colocalization quantification in HALO. In S1A Fig, the colocalized area between Q27A-L28A-T29A-gD and HVEM was significantly reduced relative to WT-gD and HVEM (S1A Fig, WT to Q27A-L28A-T29A-gD, *P* = 0.0067, one-way ANOVA). Although a decreasing trend was observed, there was no significant difference between Q27P-gD and Q27A-L28A-T29A-gD (S1A Fig, Q27P to Q27A-L28A-T29A-gD, ***P*** > 0.05, one-way ANOVA). To determine whether co-transfection of WT or mutant gD constructs with HVEM affected protein expression, we quantified the average expression of gD and HVEM across the sections. In S1B Fig and S1C, the expression levels of gD and HVEM, respectively, did not differ significantly among groups (***P*** > 0.05, one-way ANOVA). These results confirmed that mutations in aa 27–29 of gD blocked gD binding to HVEM, while a single mutation in aa 27 of gD significantly reduced but did not eliminate gD binding to HVEM.

### Construction of recombinant viruses v27 and v27‑29 expressing Q27P or Q27A-L28A-T29A, respectively, in the GFP‑McKrae background

**Fig 2:**
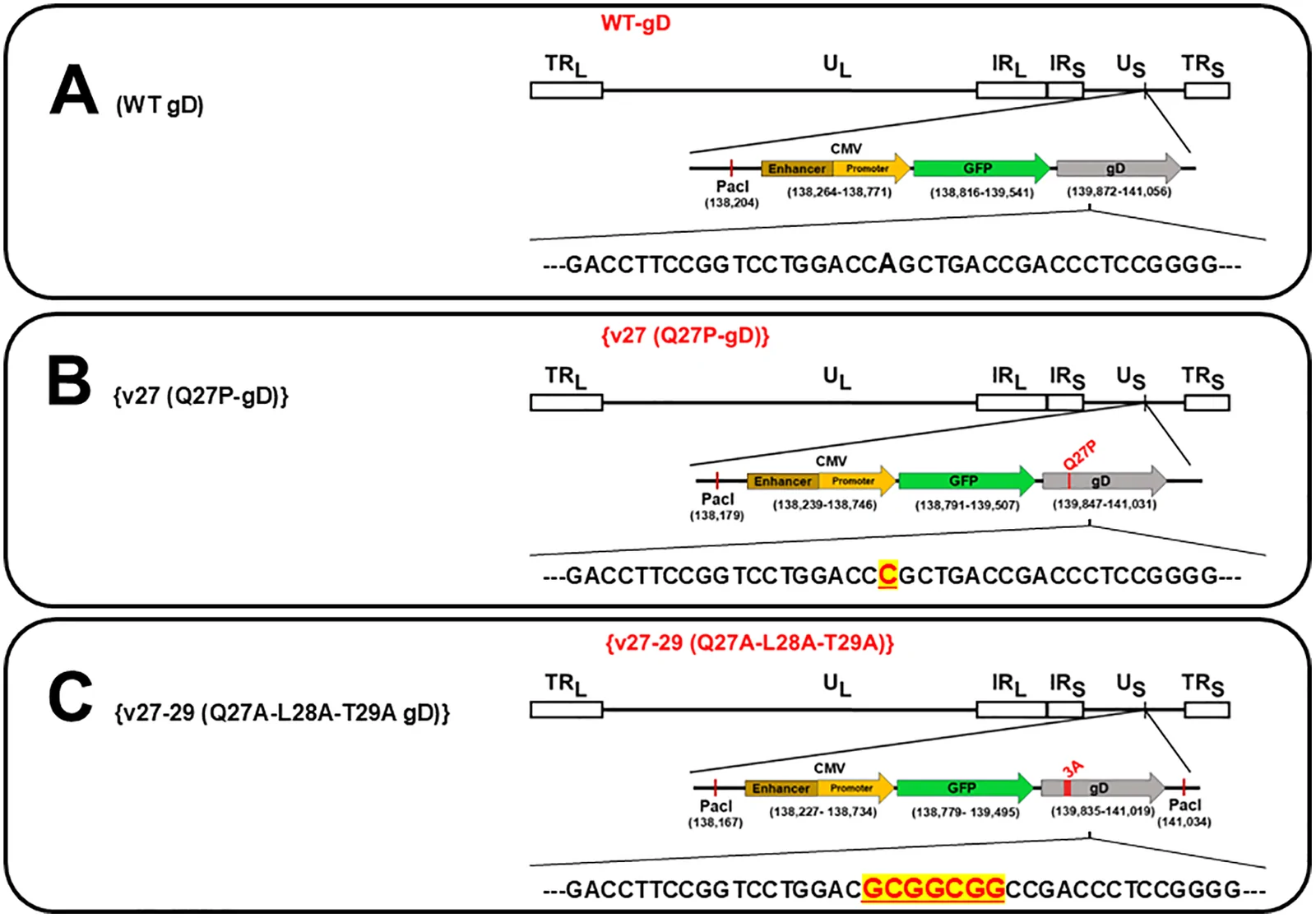
Schematic diagram of HSV-1 recombinant viruses. (A) GFP-McKrae virus genome in the prototypic orientation showing wild-type gD. TRL and IRL represent terminal and internal (or inverted) long repeats, respectively. TRS and IRS represent terminal and internal (or inverted) short repeats, respectively. UL and US represent long and short unique regions, respectively. gD gene: gray arrow. GFP: green arrow upstream of gD; (B) v27 virus. Schematic diagram of v27 virus is similar to “A”, showing Q27P mutation as a vertical red line in gD ORF; and (C) v27-29 virus. Schematic diagram of v27-29 virus as “A” but with three mutations in Q27A-L28A-T29A of gD and vertical red lines in gD showing the position of the Q27A-L28A-T29A mutations in gD ORF.

Based on mapping studies (Fig 1), we asked whether the complete loss of gD-HVEM binding affects viral replication and gene expression *in vitro*. To introduce Q27P or Q27A-L28A-T29A mutations into the viral gD coding region, plasmids containing GFP-gD mutations were synthesized (designated pUC57-GFP-Q27P-gD and pUC57-GFP-Q27A-L28A-T29A-gD), and co-transfected with WT-McKrae genomic DNA to generate mutant viruses that incorporate either single (Q27P-gD) or triple (Q27A-L28A-T29A -gD) substitutions, as we have reported [38–41]. GFP-positive recombinant viruses were plaque-purified five times to ensure genetic homogeneity, and two mutant viruses were isolated, v27 (Q27P) and v27-29 (Q27A-L28A-T29A) (Fig 2). Whole genome sequencing confirmed that the expected mutations in the gD ORF of v27 (Fig 2B) and v27-29 (Fig 2C) were present, with no unintended mutations or deletions elsewhere in the genome (Fig 2A). Complete genome sequences of v27 (GenBank accession PX763612) and v27-29 (GenBank accession PX837190) have been deposited, and are publicly available, in the NCBI GenBank. WT GFP-McKrae, as well as v27 and v27-29 viruses, are identical to their parental McKrae without GFP expression [40].

### v27 and v27-29 have similar replication kinetics in different fibroblast or epithelial cell lines

**Fig 3:**
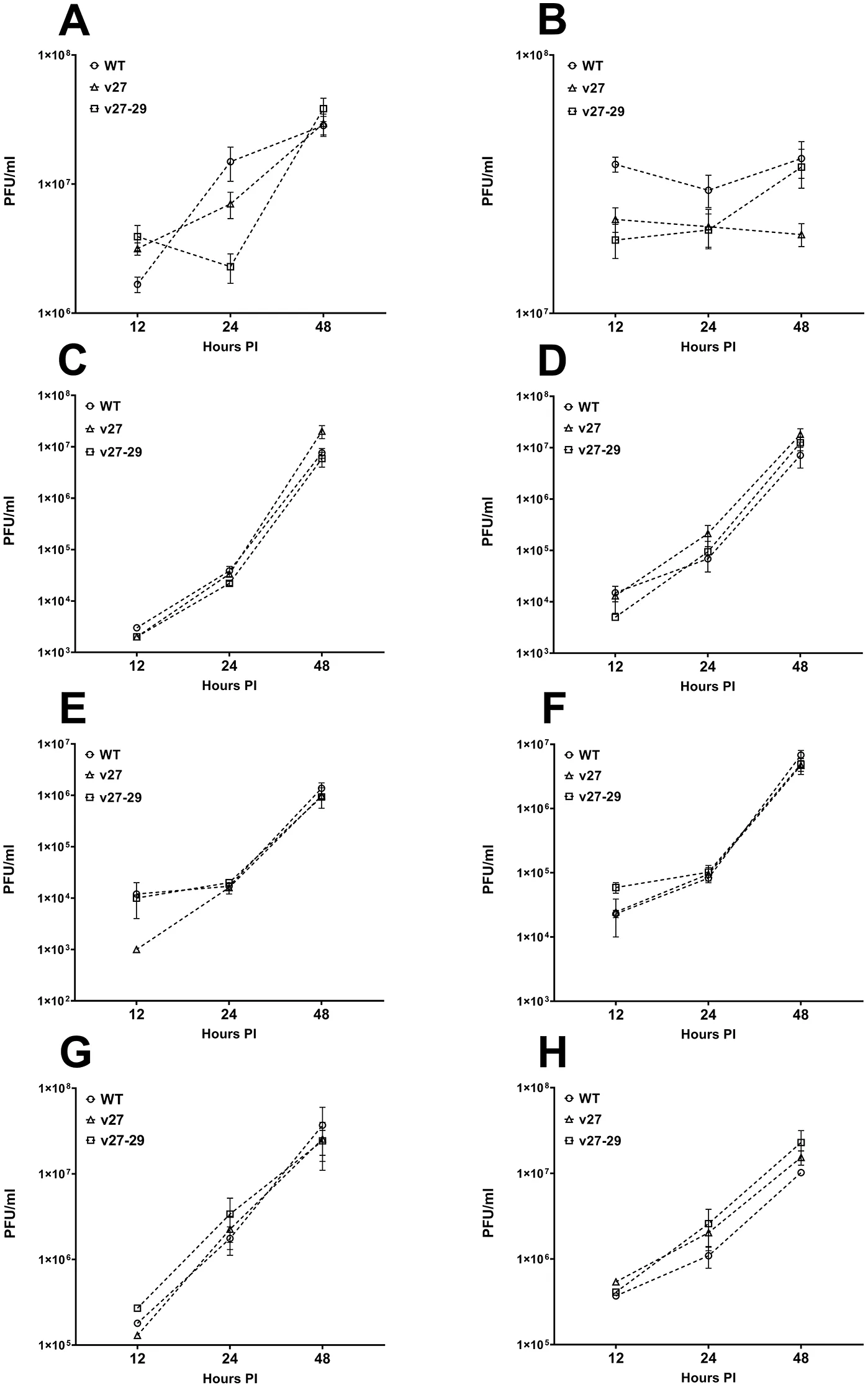
Replication of gD mutant viruses *in vitro.* Subconfluent Rabbit Skin, L929, HeLa, and MEF cell monolayers were infected in triplicate with 0.1 or 1 pfu/cell of WT, v27, and v27-29 viruses for 12, 24, and 48 hr, as described in the Materials and Methods. Panels: A and B (RS cells at 0.1 (A) and 1 (B) pfu/cell); C and D (HeLa cells at 0.1 (C) and 1 (D) pfu/cell); E and F (L929 cells at 0.1 (E) and 1 (F) pfu/cell); and G and H (MEF cells at 0.1 (G) and 1 (H) pfu/cell). The total virus was harvested at the indicated hr PI after two freeze-thaw cycles. Viral titers were quantified by standard plaque assays on RS cells. Each point represents the mean ± SEM from three separate experiments.

To determine if partial or complete blocking of gD binding to HVEM affects virus replication *in vitro*, Rabbit Skin (RS) cells, HeLa, L929, and mouse embryonic fibroblast (MEF) cells were infected with 0.1 or 1 pfu/cell of WT, v27, or v27-29 viruses for 12, 24, or 48 h. Virus titers in these infected cells were determined by standard plaque assay. No significant differences were detected between the three viruses in RS cells infected with 0.1 or 1 pfu/ cell (Fig 3A, 3B; ***P > 0.05***), HeLa cells (Fig 3C, 3D; ***P > 0.05***), L929 cells (Fig 3E, 3F; ***P > 0.05***), or MEF cells (Fig 3G, 3H**; *P* > 0.05**). These results show that insertion of Q27P or Q27A-L28A-T29A mutations in gD does not substantially affect virus replication in RS, HeLa, L929, or MEF cells. This is consistent with our current understanding of HSV receptor usage, in which alternate entry receptors can partially compensate for the loss or absence of a single receptor [42].

### Replication kinetics of v27 and v27-29 differ in neuronal cells

**Fig 4:**
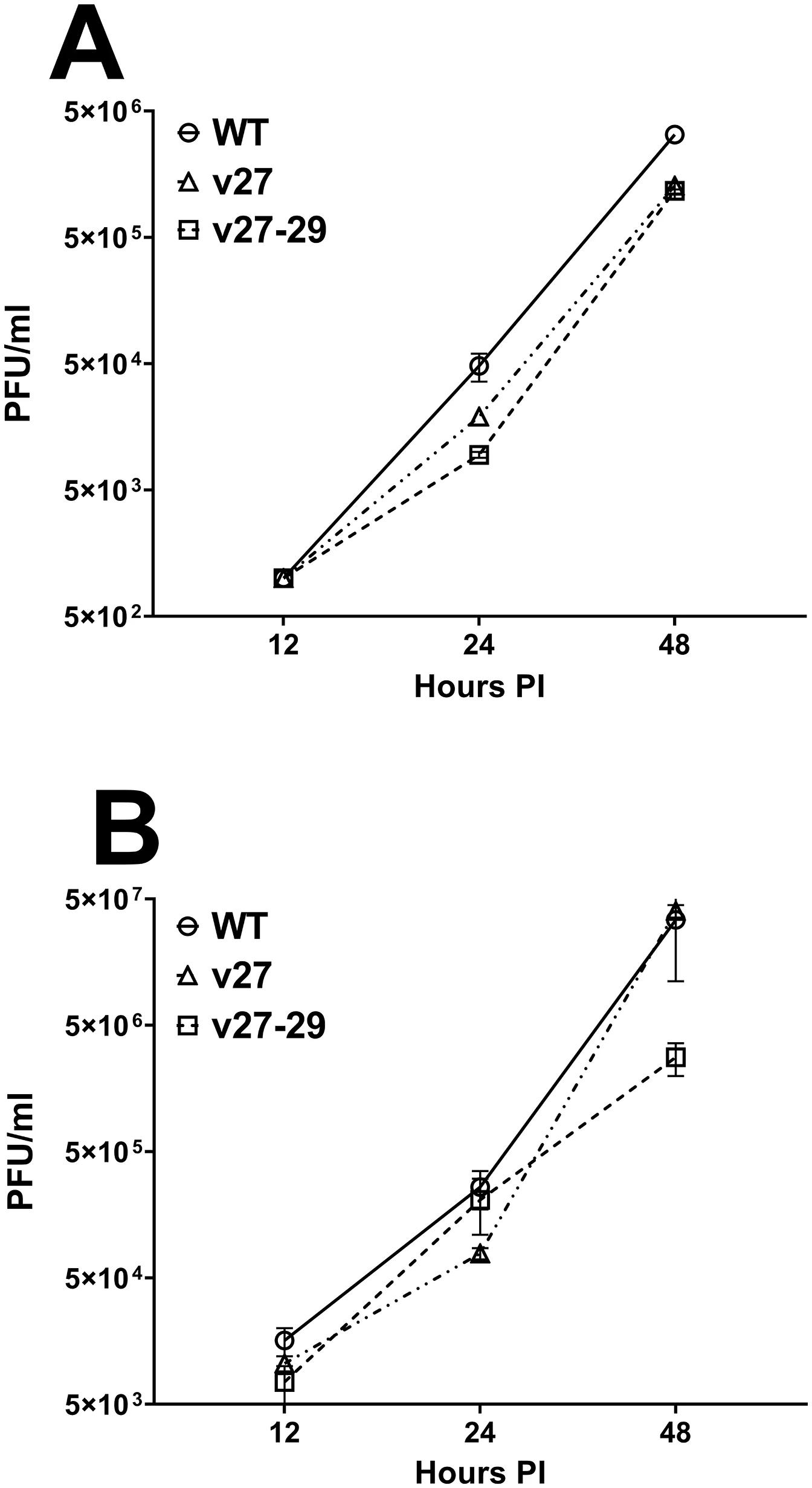
Replication of gD mutant viruses in Neuro2a cells. Subconfluent Neuro2a cell monolayers were infected in triplicate with at an MOI of 0.1 **(Panel A)** or 1 pfu/cell **(Panel B)** of WT, v27, and v27-29 viruses for 12, 24, and 48 hr, as above. Total virus was harvested at indicated times PI by two freeze-thaw cycles. Viral titers were quantified by standard plaque assays on RS cells. Each point represents the mean ± SEM from three separate experiments.

The above results, in parallel with our *in vitro* data, suggest that partial or complete reduction of gD binding to HVEM does not affect gD binding in infected fibroblasts or epithelial cells. Therefore, we asked if partial or complete blocking of gD binding to HVEM affects virus replication *in vitro*. Neuro2a cells were infected with 0.1 or 1 pfu/cell of WT, v27, or v27-29 virus for 12, 24, or 48 hr, and virus titers in infected cells were determined by standard plaque assay. At a multiplicity of infection (MOI) of 0.1 per cell, v27 and v27-29-infected Neuro2a cells had markedly lower virus yield than WT virus at 48 hr post infection (PI). However, v27 and v27-29 viruses had similar virus titers at 12 or 24 hr PI (Fig 4A, ***P > 0.05, two-way ANOVA***). At an MOI of 1, v27-29 virus yield was lower than that of v27 and WT viruses (although the difference was not significant), which had similar yields at 48 hr PI, while v27 and WT virus yields were similar (Fig 4B, ***P > 0.05, two-way ANOVA***). These late-phase differences suggest that the v27-29 mutation primarily impairs secondary infection and cell-to-cell spread rather than initial entry, consistent with the timing of gD-HVEM binding as an early event.

These results demonstrate that replication of v27 and v27-29 viruses in Neuro2a cells was less than that of the WT virus at an MOI of 0.1 (Fig 4A), while at an MOI of 1, at 48 hr PI (Fig 4B), replication of v27 and WT viruses was 13 times higher than that of v27-29. These results suggest that complete reduction of gD binding to HVEM in the v27-29 virus reduced viral replication in neuronal cell culture. This is important because HSV establishes latency and undergoes reactivation in the TG, which is also of neuronal origin.

### Absence gD and HVEM colocalization in v27-29 but not v27-infected cells

**Fig 5:**
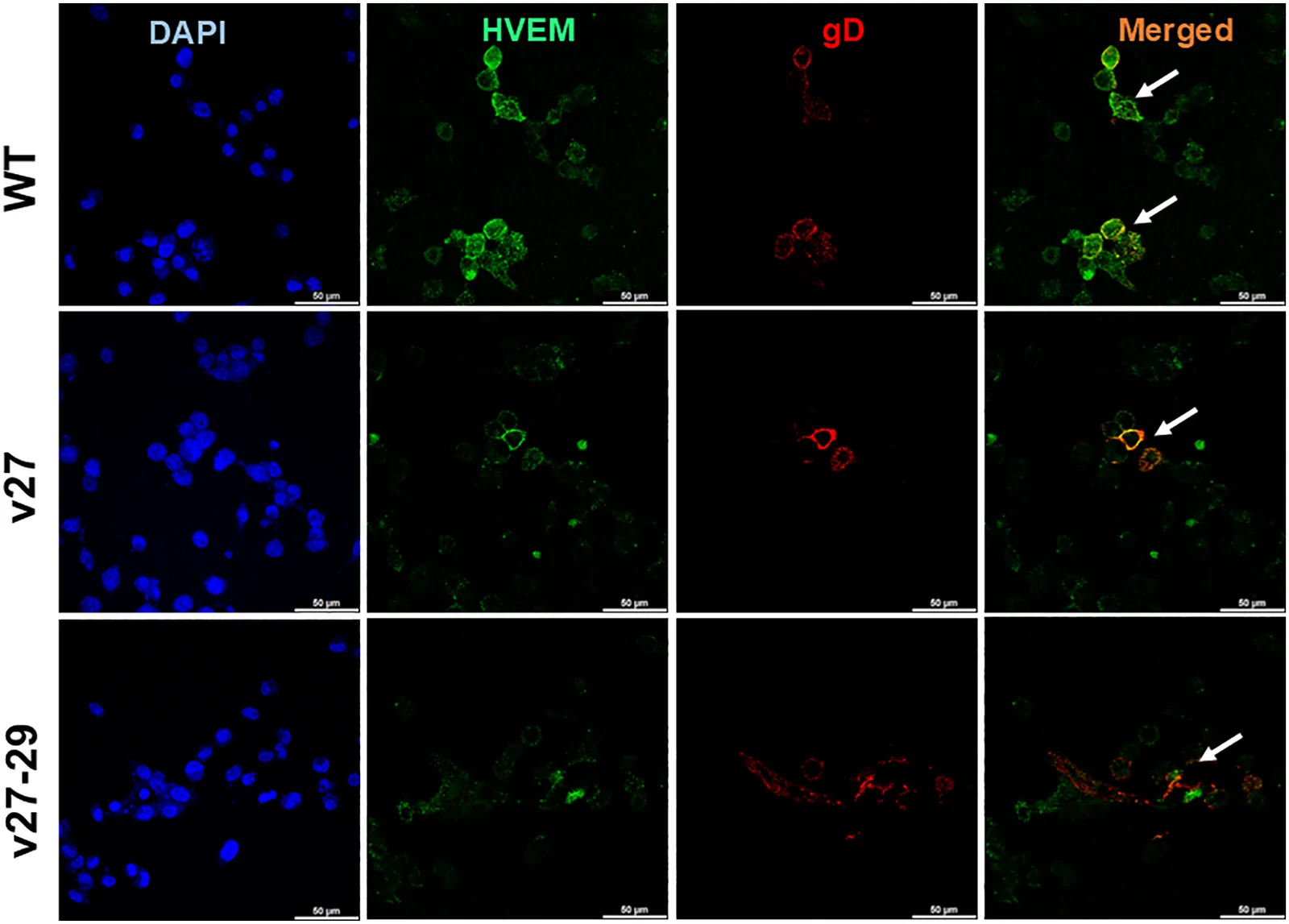
Detection of gD and HVEM expression on the surface of infected cells at 24 hr. Subconfluent Neuro2a cell monolayers were infected with 1 pfu/cell of WT, v27, or v27-29 viruses for 24 hr. Live-infected cells were immuno-stained with anti-gD and anti-HVEM antibodies and fixed with 4%PFA as described in the materials and methods. Images were acquired by confocal microscopy as described in Materials and Methods. Panels show representative infected Neuro2a cells. Scale bar, 50 μm.

Our co-IP and microscopy results from the co-transfection experiment showed reduced gD binding to HVEM with the Q27P mutant and a complete loss of binding with the Q27A–L28A–T29A mutant (Fig 1B). To determine if Q27P and Q27A-L28A-T29A mutations similarly affect cell-surface localization of gD and HVEM in infected cells, Neuro2a cells were infected with v27, v27-29, and WT viruses at 1 pfu/cell for 24 and 48 hr PI (Fig 5). Subcellular localization of live infected cells was assessed by immunostaining for HVEM and gD expression and examined by confocal microscopy. Given that the gD-HVEM interaction occurs at the membrane of infected cells, live cells were stained and then fixed to assess only surface protein expression. For analysis, 8–10 z-stacks per field were acquired, and maximum-intensity projections were generated to visualize representative expression patterns. The GFP channel was used to identify the virus-positive cells. While GFP is expressed in the viruses, HVEM and gD are stained with different colors. The signals are red and green in the figures to better see the overlap. At 24 hr PI, less HVEM expression was seen in cells infected with v27 and v27-29 than in WT-infected cells, with v27-29 showing the lowest HVEM levels (Fig 5**, HVEM panels**). Similar gD expression was seen in WT and v27-infected cells but not in v27-29-infected cells at 24 hr PI (Fig 5**, gD panels**). Merging gD and HVEM images for each virus showed that gD and HVEM co-localized on the surface of WT-infected cells. gD binding to HVEM was reduced in v27-infected cells, while no colocalization was seen in v27-29-infected cells (Fig 5**, Merge panels**).

**Fig 6:**
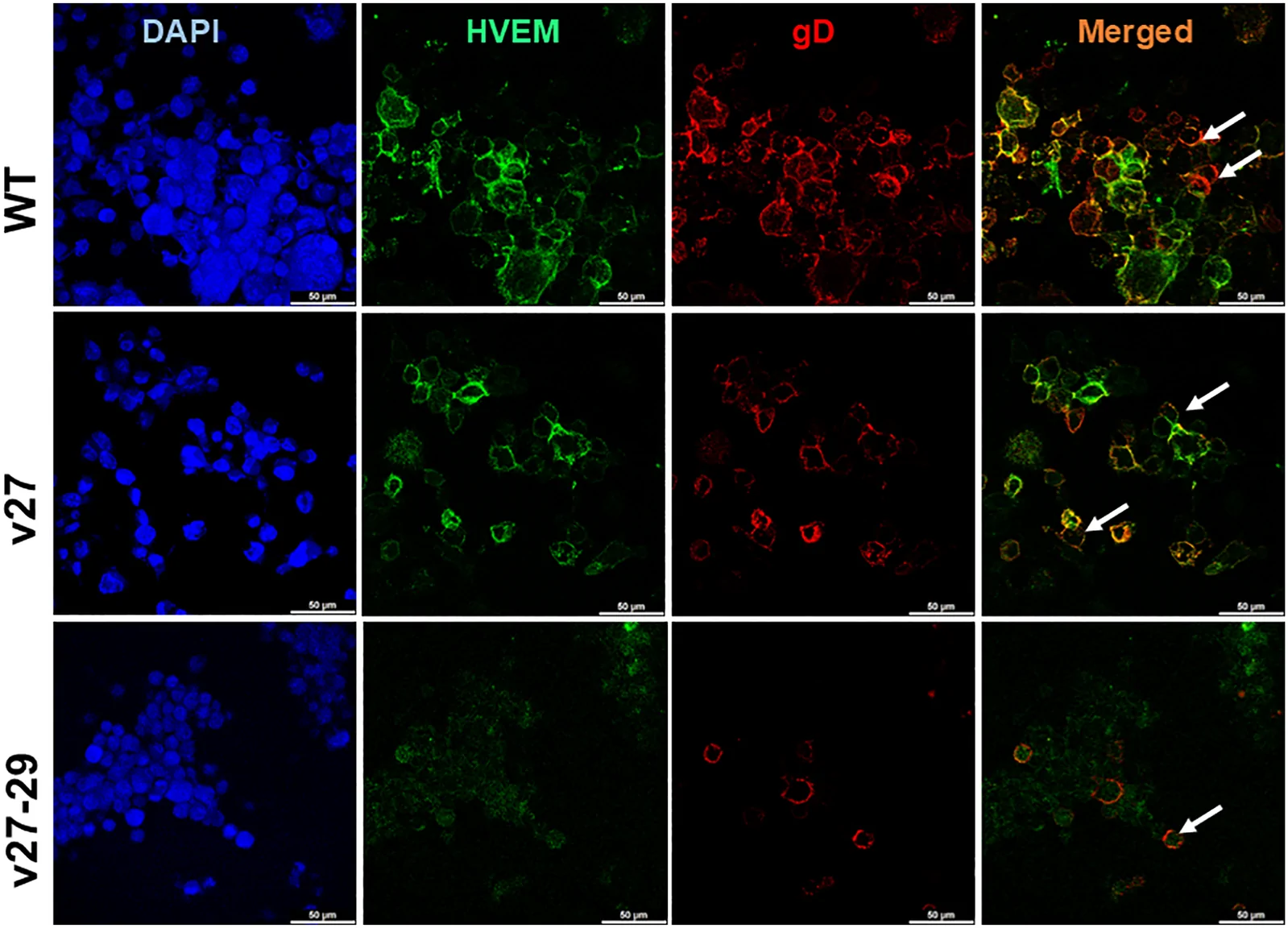
Detection of gD and HVEM expression on the surface of infected cells at 48 hr. Subconfluent Neuro2a cell monolayers were infected with 1 pfu/cell of WT, v27, or v27-29 viruses for 48 hr. Live-infected cells were immuno-stained with respective antibodies as mentioned in Fig 5. Scale bar, 50 μm.

Consistent with 24 hr results, at 48 hr PI, HVEM expression was reduced, with more reduction in v27-29-infected cells than in WT-infected cells (Fig 6**, HVEM panels**). Similarly, gD expression was reduced in the mutant groups, with more reduction in v27-29-infected cells than in WT-infected cells (Fig 6**, gD panels**). Similar to 24 hr PI results, merging gD and HVEM for each virus showed that gD and HVEM were localized on the surface of WT-infected cells; surface localization was reduced in v27-infected cells, and no binding was detected in v27-29-infected cells (Fig 6**, Merge panels**).

These results align with previous findings, suggesting that WT-gD helps maintain HVEM expression and surface abundance, whereas mutant gD does not. In addition, complete blocking of gD binding to HVEM required mutations at aa 27–29, not just aa 27, which significantly, but not completely, blocks gD binding. Thus, our results suggest that gD binding to HVEM is required to upregulate HVEM.

### Effect of single or trip gD mutations on HSV-1 transcripts

**Fig 7:**
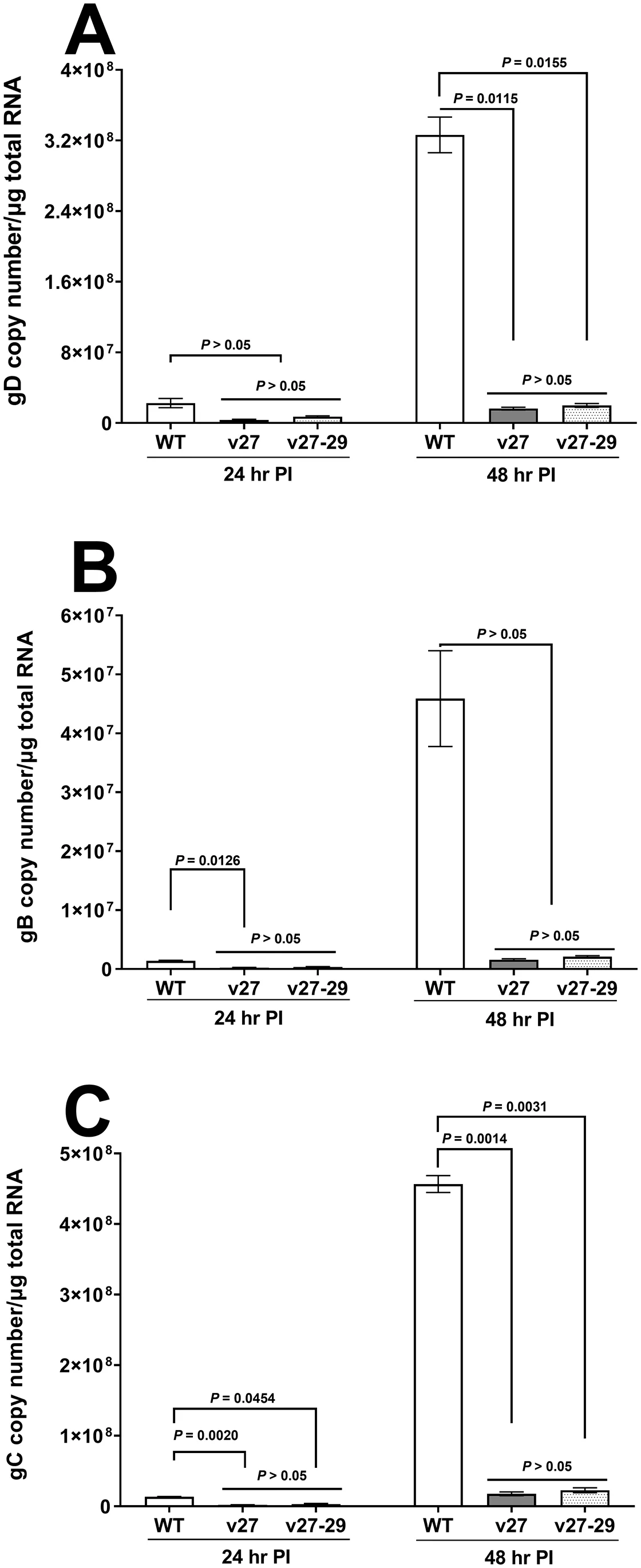
Effect of gD binding to HVEM on viral gene expression. Subconfluent Neuro2a cell monolayers were infected with 1 pfu/cell of WT, v27, or v27-29 viruses for 24 or 48 hr, as described in Materials and Methods. Total RNA was isolated from infected Neuro2a cells, and quantitative TaqMan qRT-PCR was performed on total RNA. In each experiment, the estimated relative copy number of gD, gB, or gC was calculated using standard curves generated from pAc-gD1, pAc-gB1, and pAc-gC1 plasmids. Each point represents the mean ± SEM from three separate experiments.

To further examine the effect of Q27P or Q27A-L28A-T29A mutations on the expression of viral transcripts, Neuro2a cells were infected with 1 pfu/cell of WT, v27, or v27-29 viruses for 24 and 48 hr. We measured gB, gC, and gD transcript levels by qRT-PCR to determine whether the Q27P and Q27A-L28A-T29A mutations selectively alter viral glycoprotein gene expression during infection at early and late time points. The gD copy number was lower in v27- and v27-29-infected cells than in WT-infected cells at 24 hr PI, but the differences were not statistically significant (Fig 7A**, 24 hr, *P* > 0.05**). In contrast, gD transcripts were significantly higher in WT-infected control cells at 48 hr PI than in v27 (Fig 7A**, 48 hr, *P* < 0.0115**) and v27-29-infected cells (Fig 7A**, 48 hr, *P* < 0.0155**). No differences in gD expression were in v27 and v27-29-infected cells (Fig 7A**, 48 hr, *P* > 0.05**). As with gD expression (Fig 7A), the patterns of gB (Fig 7B) and gC (Fig 7C) expression were similar at 24 hr PI. The expression of gB was significantly reduced in v27-, and v27-29-infected cells compared to the WT virus-infected cells at 24 hr PI (Fig 7A, 24 hr, P = 0.0126). At 48 hr PI, gB expression showed a reduction trend for v27 and v27-29-infected cells compared to WT virus-infected cells (Fig 7B**, 48 hr, *P* > 0.05**). Notably, expression of gC showed a significant reduction in v27-infected cells (Fig 7C**, 24 hr, *P* = 0.002**), and v27-29-infected cells (Fig 7C**, 24 hr, *P* = 0.0454**) at 24 hr PI. At 48 hr PI, expression of gC showed a significant reduction in v27-infected cells (Fig 7C**, 48 hr, *P* = 0.0014**), and v27-29-infected cells (Fig 7C**, 48 hr, *P* = 0.0031**). There was no significant difference between v27 or v27-29 viruses at any time points (Fig 7A-7C, ***P* > 0.05**). These results demonstrate that both Q27P and Q27A-L28A-T29A mutations selectively and significantly alter viral glycoprotein gene expression *in vitro,* without affecting gD protein expression. The mutant viruses showed altered mRNA expression of viral genes yet produced only slightly reduced titers, suggesting that the remaining replication steps- DNA replication, protein translation, virion assembly, egress, and infectivity of released particles- were largely preserved if the changes in one step are compensated by others.

### Effect of gD mutations on expression of its receptors (HVEM, Nectin-1, and 3-OS-HS) in infected cells

**Fig 8:**
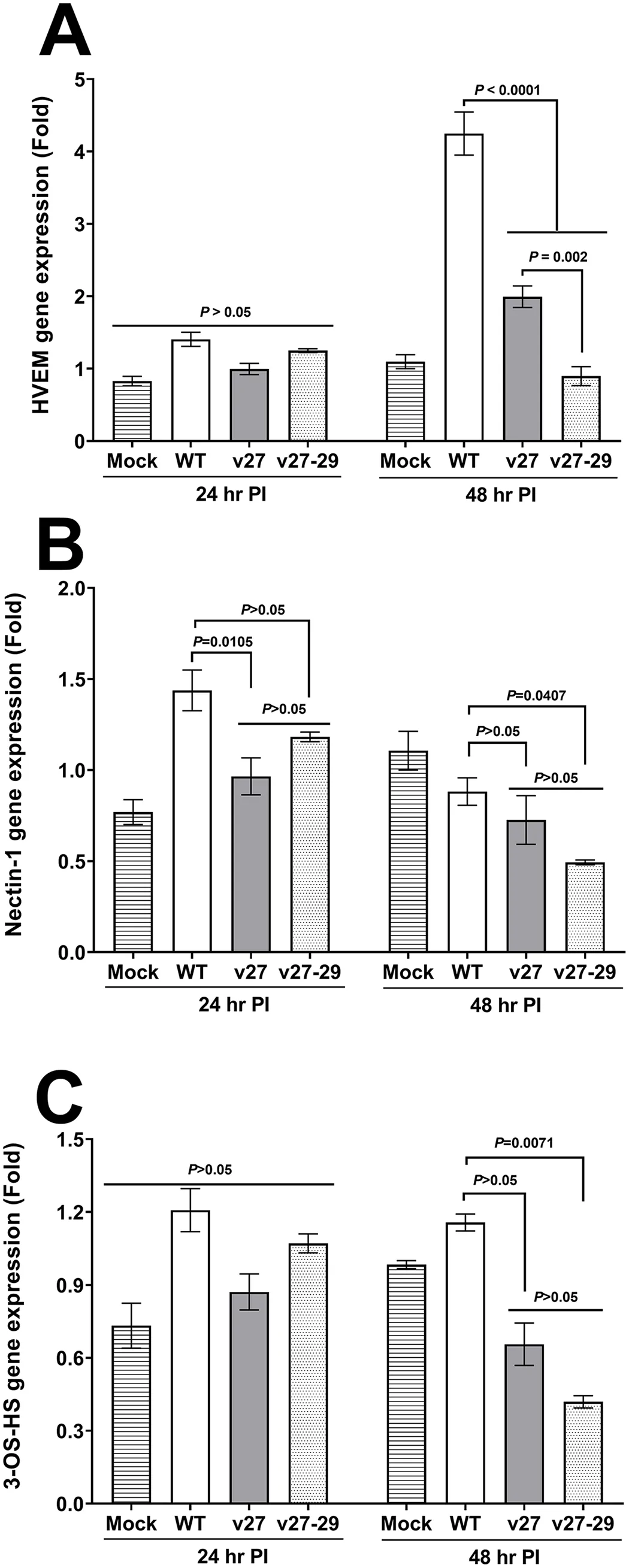
Effect of gD binding to HVEM on expression of gD-associated receptors in infected cells. RNA isolated from infected cells described in Fig 6 was used to measure expression of HVEM, nectin-1, and 3OS-SH by qRT-PCR. Relative expression levels were normalized to GAPDH and calculated relative to mock-infected cells. Each point represents the mean ± SEM from three separate experiments.

We have reported that HSV-1 upregulates HVEM but no other known HSV-1 receptor [28,29]. Because our previous studies suggested that LAT is involved in HVEM upregulation, we asked whether the absence of v27 or v27-29 binding to HVEM affects expression of the gD receptors, HVEM, nectin-1, or 3-OS-HS. Neuro2a cells were infected with 1 pfu/cell of WT, v27, or v27-29 viruses for 24 or 48 hr, and HVEM, nectin-1, and 3-OS-HS mRNA levels were measured by qRT-PCR. The results are shown as fold change in gene expression relative to mock-infected cells. At 24 hr PI, HVEM expression was similar among the three infected and mock groups (Fig 8A**, 24 hr PI, *P* > 0.05**). In contrast, expression of HVEM in v27 or v27-29-infected cells was significantly lower than that in WT-infected cells at 48 hr PI (Fig 8A**, *P* < 0.0001, 48 hr PI**). Notably, HVEM expression was lower in v27‑29-infected cells than in v27-infected cells at 48 hr PI **(**Fig 8A**, *P* = 0.002)**. Nectin-1 expression was markedly lower in v27- (Fig 8B**, *P* = 0.0105**) infected cells at 24 hr PI, than in WT-infected cells. At 48 hr PI, nectin-1 levels were significantly lower in v27-29-infected cells than in v27- or WT-infected cells (Fig 8B**, *P* = 0.0407**). No significant differences in 3-OS-HS expression were observed among the three infected groups at 24 hr PI (Fig 8C**, 24 hr, *P* > 0.05**), whereas its expression was significantly lower in v27-29 than in WT (Fig 8C**, *P* < 0.0071**) infected cells at 48 hr PI.

These results further demonstrate that both Q27P and Q27A-L28A-T29A mutations affect host receptor expression, with the greatest reduction in receptor expression observed in the v27-29 triple-mutant virus. In contrast to previous reports suggesting that the Q27P mutation in HSV gD enables the virus to adapt to nectin‑1 in the absence of HVEM [24,25]. We found that altering gD aa 27–29 disrupts and may even impair the virus’s ability to use both HVEM and nectin‑1 as entry receptors.

### Effect of blocking gD binding to HVEM on other members of the TNF receptor superfamily

**Fig 9:**
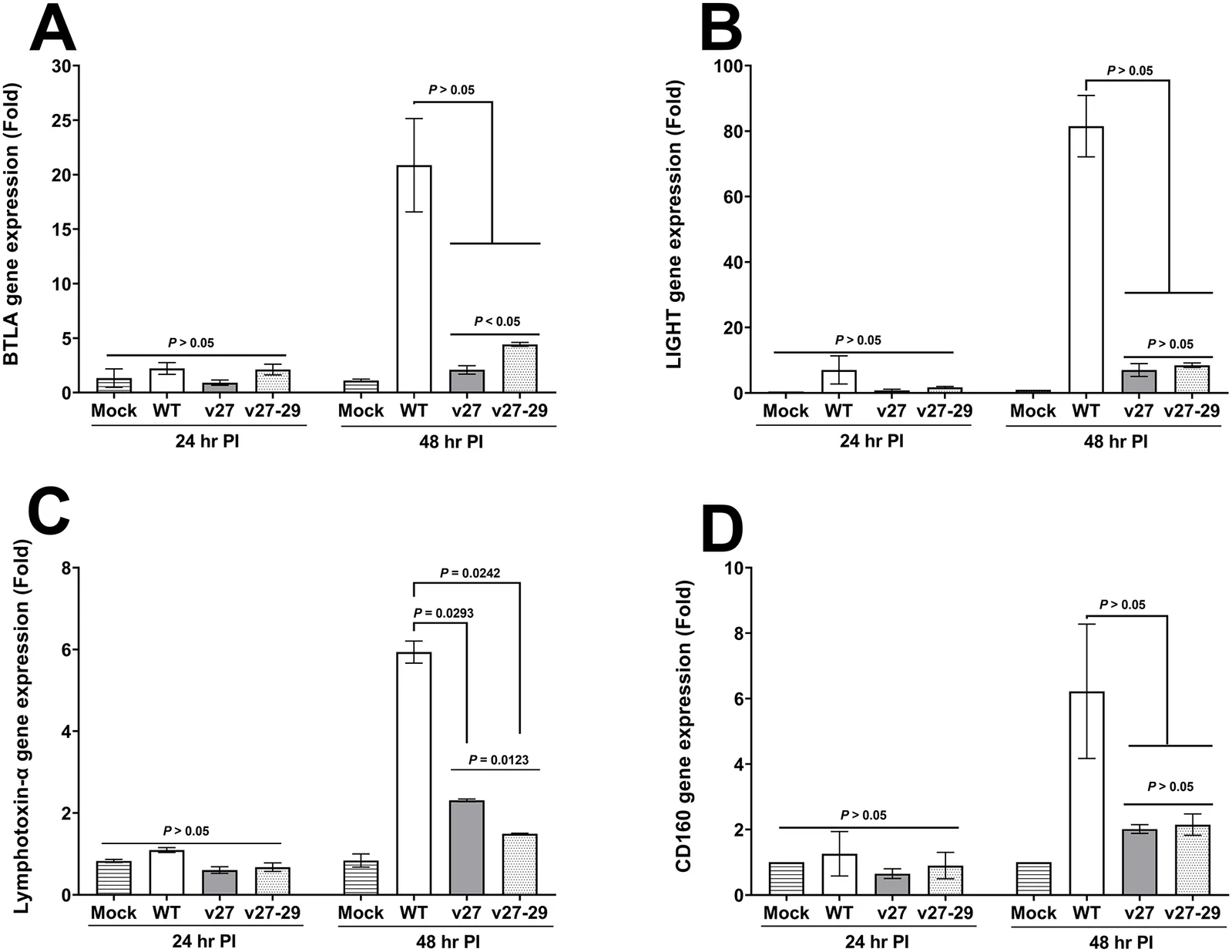
Effect of gD binding to HVEM on TNF superfamily expression in infected cells. RNA isolated from infected cells (as described in Fig 6) was used to measure expression of BTLA, LIGHT, Ltα, and CD160, as described in the Materials and Methods. Relative expression was normalized to GAPDH and calculated relative to mock-infected cells. Each point represents the mean ± SEM from three separate experiments.

In addition to HVEM, LIGHT (TNFSF14), lymphotoxin-α (LTα), CD160, and BTLA are also members of the TNF receptor superfamily [32,34]. They act as a molecular switch between proinflammatory and inhibitory signaling to regulate homeostasis [43,44]. In addition to gD, HVEM is activated by LIGHT (TNFSF14), lymphotoxin-α (LTα), CD160, and BTLA [26,43,44]. To determine if Q27P and Q27A-L28A-T29A mutations also affect HVEM-ligand expression, Neuro2a cells were infected with WT, v27, or v27-29 viruses at 1 pfu/cell, and levels of BTLA, LIGHT, lymphotoxin-α, and CD160 mRNA were measured by qRT-PCR at 24 and 48 hr PI. At 24h, the relative expression of BTLA and LIGHT was similar among cells infected with the three viruses (Fig 9A-9B**, *P* > 0.05)**. A similar trend in expression was observed for lymphotoxin-α (Fig 9C**, 24 hr, *P* > 0.05**) and CD160 (Fig 9D**, 24 hr, *P* > 0.05**).

At 48 hr PI, BTLA and LIGHT expression were both increased in WT cells than in v27- and v27-29-infected cells, although the difference was not significant (Fig 9A**-**9B**, *P* > 0.05**), but v27- and v27-29-infected cells did not differ significantly (Fig 9A-9B, ***P* > 0.05**). At 48 hr PI, lymphotoxin-α expression in WT-infected cells was significantly increased compared to v27- (Fig 9C**, *P* = 0.0293**) and v27-29-infected cells (Fig 9C-9D**, *P* = 0.0242**). Notably, at 48 hr PI, v27‑29-infected cells expressed less lymphotoxin-α than did v27-infected cells (Fig 9C, ***P* = 0.0123**). In addition, CD160 levels showed a similar reduction in the mutant viruses compared to the WT virus (**although the differences were not significant;** Fig 9D, **48 hr, P > 0.05**). The expression was similar between v27- and v27-29-infected cells (Fig 9D**, 48 hr, *P* > 0.05**). These results suggest that Q27A-L28A-T29A mutations profoundly affect host receptor expression and HVEM-ligand levels.

## Discussion

The HSV-1 gD gene is essential, with homologs that are highly conserved in different HSV-1 strains (i.e., KOS, F, McKrae, 17, H129, SC, ANG) [45–48]. gD plays a key role in virus infection and pathogenesis by binding to the cellular HVEM receptor, thereby influencing HSV‑1 latency and reactivation in corneal epithelium, immune cells, and neurons. gD is a major inducer and target of humoral and cell-mediated immune responses following infection [49,50], mediated by its binding to HVEM, nectin-1, and 3-OS-HS [6,24,37,51–53]. In previous studies, mutations at two sites within the gD N-terminal loop were shown to alter receptor usage [24,37,53]. Substitution of gD-L25 with proline, or gD-Q27 with proline or arginine, permitted the use of the nectin-2 receptor for virus entry [37,54]. Single-amino-acid substitutions in gD, specifically Q27P and Q27R, allow the virus to resist gD-mediated interference [37]. These mutations impair entry via the HVEM receptor while enabling or enhancing entry via other receptors, such as nectin-2, thereby altering the virus's ability to infect different cells. Introduction of the Q27P mutation into gD interfered with its ability to interact with HVEM, resulting in a 10-fold greater affinity for nectin-1 [24]. A subsequent study showed that a gD-1 (delta290-299t) mutant similarly blocks HVEM entry while preserving nectin-1 function [53].

Because most of the above studies used less virulent viruses, we generated recombinant viruses using the highly virulent ocular HSV-1 strain McKrae. Before creating mutant gD viruses in McKrae that do not bind to HVEM, we performed mapping studies and found that substitution of glutamine with proline at aa 27 of gD significantly, but not completely, reduced its binding to HVEM, whereas a Q27A-L28A-T29A triple mutation completely abolished this interaction. Despite these substantial changes in receptor engagement, the replication kinetics of v27 and v27-29 were similar to those of the WT virus in four different mouse, rabbit, and human epithelial cell lines. However, replication of the two viruses differed from WT virus in Neuro2a cells, which share a neuronal origin. Similar growth curves indicate that alternate gD receptors, such as nectin-1 and 3-OS-HS, can compensate for reduced HVEM binding to sustain productive replication. Although both nectin-1 and HVEM are expressed in cells and neurons, HSV-1 preferentially uses nectin-1 for both neuronal and epithelial cell entry. For example, antibodies against nectin-1, but not against HVEM, inhibit HSV-1 entry into cultured neurons [55] and epithelial cells [56]. Additionally, mutations in gD that inhibit its binding to nectin-1 prevent infection of neurons, whereas mutations that eliminate crucial HVEM binding sites in gD do not [57]. Our results are consistent with previous findings that HSV-1 mutants that cannot bind HVEM (e.g., Rid1) replicate similar to the WT virus in nectin-1-expressing cells, such as Vero and HEp-2, but have profound entry defects in HVEM-dependent Chinese hamster ovary cells [58]. These results indicate that disrupting a single receptor pathway often has only minor effects on acute viral titers when other receptors remain available. In addition to HVEM, v27-29-infected cells also had reduced nectin-1 and 3-OS-HS expression at 48 hr PI. Similar to our previous study [28], Neuro2a cells infected with WT virus showed higher HVEM expression than those infected with v27 or v27-29. However, a similar pattern of nectin-1 and 3-OS-HS expression was not detected. Reduced replication of v27 and v27-29, especially v27-29, in Neuro2a cells positively correlated with viral gene expression, as gB, gC, and gD expression were significantly reduced in infected Neuro2a cells.

Reduced expression of HVEM, nectin-1, and 3-OS-HS in Neuro2a cells infected with v27 and v27-29 suggests a broader regulatory role for gD in shaping the neuronal receptor landscape. HVEM expression was not increased in v27- or v27-29-infected cells compared with the higher levels observed in WT-infected cells, parallels the degree of impairment in gD-HVEM binding observed in pull-down assays, supporting a direct mechanistic link between physical interaction and steady-state receptor expression. Consistent with our previous works [28,29], we found that LAT(+) viruses upregulate HVEM transcription in TG neurons via NF-κB signaling, while *HVEM*^*-/-*^ mouse studies confirmed that host HVEM promotes viral reactivation independent of LAT, and that LAT itself upregulates neuronal HVEM to enhance reactivation efficiency. Concomitant downregulation of nectin-1 and 3-OS-HS suggests that loss of gD-HVEM binding may modulate cellular pathways that govern expression or stability of multiple viral entry receptors, potentially through HVEM-associated signaling cascades.

Similar to gD, HVEM is a ligand for BTLA, LIGHT, and LTα and can block each in a ligand- and epitope-specific manner [42,44]. gD directly competes with BTLA for HVEM binding, but not with LIGHT or LTα, which reflects their overlapping (HVEM) and non-overlapping (LIGHT, LTα) binding footprints on HVEM [42]. Our results suggest that gD binding to HVEM is required to upregulate HVEM and the HVEM pathway. In contrast, we previously reported that LAT upregulates HVEM but not other members of the TNF superfamily [28]. The absence of major differences in viral replication across these diverse cell lines confirmed that the mutant viruses retain comparable growth capacity *in vitro* under non-selective conditions. Subsequently, the lack of upregulation of the HVEM ligands BTLA, LIGHT, Ltα, and CD160 suggests that canonical HVEM signaling was not enhanced in the absence of gD binding, compared with WT gD expression, where significant upregulation of these genes was observed. Both complete loss and partial reduction of gD binding to HVEM were associated with significantly reduced transcription of BTLA, LIGHT, Lta, and CD160 compared with WT. HVEM is activated by binding to LIGHT and Ltα and binds to CD160 and BTLA [32,43,44]. In this context, WT-gD may function not only as a viral ligand that exploits HVEM for entry but also as a modulator of host receptor expression that promotes a receptor milieu favorable for recurrent infection. In contrast, gD mutants that do not efficiently engage HVEM, fail to maintain this permissive state. These findings suggest that the transcriptional regulation of this ligand-receptor axis is differentially influenced by the two mutant viruses, which may reflect distinct effects of the viral mutations on immune modulatory signaling.

Our pull-down assays using transfected cells showed that a single mutation in gD aa 27 reduced its binding to HVEM by approximately 80%, while mutations in gD aa 27–29 completely blocked its binding to HVEM. This discrepancy between our results and those of previous studies [24,37] is likely due to the use of different binding assays. Thus, to confirm our pull-down assays showing partial-to-complete gD binding to HVEM, we asked whether cells infected with the v27 and v27-29 viruses showed similar results. We examined cell-surface expression and gD binding in infected cells using both unfixed and fixed cells. As expected, WT-gD from WT-infected virus binds to HVEM on the surface of infected cells when HVEM and gD staining were co-localized. In v27-infected cells, we saw significantly less staining of co-localized HVEM and gD, while gD and HVEM did not co-localized in v27-29-infected cells. These results support our overall hypothesis that aa 27–29, but not aa 27 alone, is required to completely block gD binding to HVEM. These results also suggest that gD-HVEM engagement not only mediates receptor-dependent entry but also positively regulates HVEM abundance at the neuronal surface, to potentially establish a feed-forward loop that favors efficient gD-HVEM signaling during infection and reactivation.

The results of this study support a model in which specific gD aa that governs HVEM binding, also fine-tune HSV-1 interactions with neuronal receptors, without significantly altering lytic replication *in vitro*. In this context, v27 and v27-29 viruses provide useful tools to determine how specific changes in gD-HVEM binding alter latency and reactivation dynamics *in vivo*. Future *in vivo* studies will be needed to determine whether targeting the gD Q27-T29 region, or downstream HVEM-dependent signaling, can selectively reduce HSV-1 reactivation without substantially compromising control of primary infection.

## Materials and methods

### Cells and viruses

RS cells were used to prepare virus stocks, culture mouse tear swabs, and determine viral growth kinetics. RS cells were grown in Eagle’s minimal essential medium supplemented with 5% fetal bovine serum. Triple plaque-purified HSV-1 WT, v27, and v27-29 viruses were used in this study. HEK293T, HeLa, L929, Neuro2a, and Mouse embryonic fibroblast (MEF) cells were cultured in DMEM medium supplemented with 10% FBS.

### Co-immunoprecipitation

We generated three gD constructs: a full-length WT-gD ORF with a myc tag (WT-gD), a single-point mutant plasmid containing a Q27P substitution with a myc tag, and a triple mutant containing Q27A-L28A-T29A substitutions with a myc tag. We also generated a full-length mouse HVEM ORF with dual Flag tags (HVEM-2xFlag). All constructs were cloned into the pcDNA3.1 vector as we described previously [59]. HEK 293T cells were seeded in 10 cm tissue culture dishes at 4.4 x10^6 cells/dish. Next day, cells were transfected with 10ug mouse HVEM 2XFlag plasmid and 5 ug of one of the gD constructs. Cells were lysed in 0.9 ml lysis buffer (100mM NaCL, 20mM Tris HCL, pH 7.4, 1% NP-40, protease inhibitor cocktail (Roche Life Sciences, Penzberg, Germany) 24 hr post-transfection. Lysates were incubated on ice for 30 min and then cleared by centrifugation for 10 min at 13,000 rpm at 4°C. 70 uL lysate was saved for input, and 70 uL 2X-LDS sample buffer (NuPage LDS sample buffer, Invitrogen cat # NP0007) supplemented with 5% mercaptoethanol (Fluka) was added. Lysate was added to Protein G Dynabeads (Dynabeads Protein G, Invitrogen, cat # 10003D) or Anti-DYKDDDDK magnetic agarose beads (Pierce, cat # A367997) and incubated overnight with rotation at 4 °C. Samples were then washed 3 times, 5 minutes each, in lysis buffer. 50 uL LDS sample buffer was added to the washed beads to elute bound proteins. Samples were incubated at 75˚C for 10 minutes.

Proteins were separated by SDS-PAGE, followed by transfer to nitrocellulose membrane. Membrane was blocked using 5% non-fat dry milk in PBST and incubated with primary antibodies (Mouse anti-gD Abcam cat #: Ab 6507, 1:1000 dilution for WB; Mouse anti Flag M2 Sigma Cat # F1804, 1:1000 dilution; Purified Rat anti DYKDDDK clone L5, Biolegend cat # 637301, 1:500) followed by HRP conjugated secondary antibodies (Goat anti mouse IgG secondary antibody, HRP Invitrogen cat # 62–6520; Biolegend Goat anti Rat IgG clone Poly 4054 cat # 405405). Images were acquired using FluorChem E (Protein Simple, San Jose, CA).

### Construction of gD mutant viruses

Previously, we constructed a recombinant HSV-1 virus expressing GFP upstream of gD in a WT-McKrae background, referred to as GFP-McKrae [40]. Based on WT-McKrae nucleotides 136441–139321, we synthesized two plasmids: one with a single aa mutation in gD aa 27 (Q27P); and the second with three aa mutations (Q27A-L28A-T29A). Both plasmids contain a complete GFP sequence under the control of a cytomegalovirus (CMV) promoter, flanked by two unique Pac1 sites (GenScript, Piscataway, NJ). These constructs were inserted into the pUC57 Pac1 site, and the resulting plasmids were designated pUC57-Q27P-gD and pUC57-Q27A-L28A-T29A-gD. Recombinant viruses v27 (Q27P mutation) and v27-29 (Q27A-L28A-T29A) were generated by co-transfecting pUC57-Q27P-gD or pUC57-Q27A-L28A-T29A-gD together with infectious McKrae DNA using the calcium phosphate method as described [40,60–62]. Viruses from the co-transfection were plated, and isolated plaques were picked and screened for GFP expression as we described [40]. Selected plaques with GFP gene expression were plaque-purified five times. Presence of the expected gD mutations was confirmed by PCR and next-generation sequencing. Complete genome sequences of v27 (**GenBank accession PX763612**) and v27-29 (**GenBank accession PX837190)** have been deposited in GenBank.

### Kinetics of virus replication in tissue culture

RS, L929, HeLa, MEF, and Neuro2a cell monolayers at 70–80% confluency were infected with 0.1 and 1 pfu/cell of WT, v27, or v27-29 virus. Virus was harvested at 12, 24, or 48 hr PI by subjecting cell monolayers to two freeze-thaw cycles. Virus titers were determined by standard plaque assay on RS cells, as described [63].

### Co-expression of gD and HVEM in infected cells

Neuro2a cells were cultured on glass coverslips, and monolayers at 80% confluency were infected with WT, v27, or v27-29 viruses at 1 pfu/cell for 24 or 48 hr. Following infection, cells were washed twice with phosphate-buffered saline (PBS) and blocked with blocking solution (2% BSA + 2% goat serum +2% milk in 0.1% PBS) for 30 minutes at room temperature. Live cells were incubated with rabbit anti-gD (Thermo Fisher Scientific, PA1–30233; 1:50) and Armenian hamster anti-mouse HVEM (Thermo Fisher Scientific, 14-5962-82; 1:100) antibodies in blocking buffer for 1 hr at room temperature. Cells were washed 3 times with 1 × PBS and incubated with goat anti-rabbit Alexa Fluor 647 (Thermo Fisher Scientific, A32733TR; 1:100) and goat anti-Armenian hamster Alexa Fluor 555 (Thermo Fisher Scientific, A78964; 1:100) secondary antibodies for 1 hour at 25 °C. Following secondary incubation, slides were washed 3 times with 1 × PBS and fixed with 4% paraformaldehyde (PFA) for 10 minutes at room temperature. Samples were mounted using ProLong Gold Antifade reagent with DAPI (Invitrogen, P36931). Images were acquired on a Leica Stellaris 8-STED super-resolution confocal microscope (Leica Microsystems, Buffalo Grove, IL). Images were processed using LAS X Office 1.4.4 software. Cells with fluorescent signals were initially identified at 20 × magnification with 16-bit resolution. Images of virus-positive regions were captured at a resolution of 1024 × 1024 pixels, with a scanning speed of 400, bidirectional scanning enabled, and a line average of 4. The GFP channel was used to identify the virus-positive cells. While GFP is expressed in the viruses, HVEM and gD are stained with different colors. The signals are red and green in the figures to better see overlapping. For analysis, 8–10 z-stacks per field were acquired, and maximum-intensity projections were generated to display representative expression patterns.

### HALO analysis for colocalization

Immunofluorescence images were analyzed using HALO image analysis software (version 4.0.5; Indica Labs). Confocal fluorescence images were acquired using a Leica STELLARIS 8 confocal microscope with a 63 × objective. Z-stack images were imported into HALO for quantitative analysis. The DAPI channel was used to assist with nuclear identification and cell localization. Fluorescence-positive areas for each marker were defined using intensity thresholds established from negative-control slides and then applied uniformly to all experimental images. Marker-positive and co-localized regions were quantified using the HALO Area Quantification FL module. Co-localized areas were identified by overlapping marker-positive masks and were used to measure the co-expression area and mean fluorescence intensity within the flagged regions. Colocalization analysis was performed using the phenotype function in the HALO Area Quantification FL module. Positive fluorescence areas for each channel were first defined using thresholds established from negative-control images. A colocalized region was then identified by applying a phenotype criterion requiring the area to be positive for both fluorescence channels. Regions meeting this dual-positive criterion were flagged as colocalized areas, and the total colocalized area and mean fluorescence intensity within these regions were quantified for each image.

### RNA extraction, cDNA synthesis, and TaqMan RT-PCR

Neuro2a cell monolayers at 80% confluency were infected with 0.1 or 1 pfu/cell of WT, v27, or v27-29 viruses. Infected cells were harvested at 24 or 48 hr PI. Total RNA was extracted using chloroform (Ambion, Austin, TX), purified using RNeasy columns (Qiagen, Hilden, Germany), and cDNA was synthesized as described previously [63]. Quantitative real-time PCR (qRT-PCR) was performed using custom TaqMan probe and primer sets for HSV-1 gB, gC, and gD, and the expression of HVEM, nectin-1, heparan sulfate-3-O-sulfotransferase (3-OS-HS), LIGHT, BTLA, CD160, and lymphotoxin-α was assessed in infected cells. In all experiments, GAPDH was used to normalize transcript levels. Each primer-probe set contained two unlabeled PCR primers and a FAM-labeled TaqMan minor groove binder probe, formulated as a single mixture. All cellular amplicons spanned intron-exon junctions to eliminate potential amplification of genomic DNA. The following assays were used in this study: 1) HVEM, ABI assay I.D. Mm00619239_m1 – amplicon length = 65 bp; 2) LIGHT, ABI Mm00444567_m1 – amplicon length = 68 bp; 3) BTLA, ABI Mm00616981_m1 – amplicon length = 71 bp; 4) CD160, ABI Mm00444461_m1 – amplicon length = 61 bp; 5) lymphotoxin-α, ABI Mm00440228_gH (amplicon size, 59 bp); 6) nectin-1, ABI Mm00445392_m1 (amplicon size, 71 bp); 7) 3-OS-HS, ABI Mm00479621_m1 (amplicon size, 65 bp); 8) HSV-1 gC, ABI ARCE4XE – amplicon length = 122 bp; and 9) GAPDH used to normalize transcripts, ABI Mm999999.15_G1 – amplicon length = 107 bp. Levels of gB and gD RNA from infected Neuro2a cells were determined using custom-made: 1) gB-specific primers (forward - 5’-AACGCGACGCACATCAAG-3’; reverse - 5’-CTGGTACGCGATCAGAAAGC-3’; and probe - 5’-FAM-CAGCCGCAGTACTACC-3’), with a primer amplicon length of 72 bp; and 2) a gD primer and probe set with: forward primer, 5’-GCGGCTCGTGAAGATAAACG-3’; reverse primer, 5’-CTCGGTGCTCCAGGATAAACTG-3’; and probe, 5’-FAM-CTGGACGGAGATTACA-3’, and amplicon length = 59 bp. Relative gB, gC, and gD copy numbers were calculated using standard curves generated from plasmids pAc-gB1 [64], pAc-gC1 [65], and pAc-gD1 [49].

### Statistical analysis

For all statistical tests, *p*-values less than or equal to 0.05 were considered statistically significant and are indicated by a single asterisk (*), and *p*-values less than or equal to 0.001 are indicated by double asterisks (**). Two-way analysis of variance (ANOVA) was used to compare differences among three or more experimental groups. All experiments were repeated at least three times to ensure accuracy.

**Original data:** All raw data to generate the figures and the Western blot of Fig 1 are within the Supporting Information files.

## Supporting information

S1 Fig gD mutations at aa 27–29 abolish binding to HVEM in vitro. HEK293T cells were transfected with plasmids encoding mouse HVEM-2xFlag and either WT-gD, Q27P-gD, or Q27A-L28A-T29A-gD tagged with Myc for 72 hr, as previously described in Fig 1. Confocal images were analyzed for colocalization using HALO software. (A) Quantification of colocalized area (*μm*^2^) for WT-gD, Q27P-gD, or Q27A-L28A-T29A-gD with HVEM. Each point represents the mean ± SEM from 5, 6, and 8 fields from HEK293T cells transfected with WT-gD, Q27P-gD, or Q27A-L28A-T29A-gD and HVEM, respectively. (B, C) Mean expression of gD and HVEM in cells transfected with WT-gD, Q27P-gD, or Q27A-L28A-T29A-gD and HVEM.
(PDF)

S2 Fig Colocalization of gD and HVEM expression on the surface of infected cells. Subconfluent Neuro2a cell monolayers were infected with 1 pfu/cell of WT, v27, or v27-29 virus for 48 hr, as described in Fig 6. Live-infected cells were immunostained with anti-gD and anti-HVEM antibodies, then fixed with 4%PFA, as described in the Materials and Methods. Colocalized area (*μm*^2^) of gD and HVEM was quantified in WT-, v27- or v27-29-infected cells. Each point represents the mean ± SEM from 15, 10, and 26 fields from WT-, v27-, and v27-29-infected cells.
(PDF)

S3 Fig Rawgel. (PNG)

S1 File Data. (PDF)
