# Supplementary material for: Complete disruption of gD binding to HVEM alters virus replication and host receptor homeostasis
Source: PLoS Pathog. 2026 Sep 8;22(9):e1014566. doi: 10.1371/journal.ppat.1014566 (PMC13581205; doi:10.1371/journal.ppat.1014566)
Supplement: Supplementary file 2 [file ppat.1014566.s002.pdf]

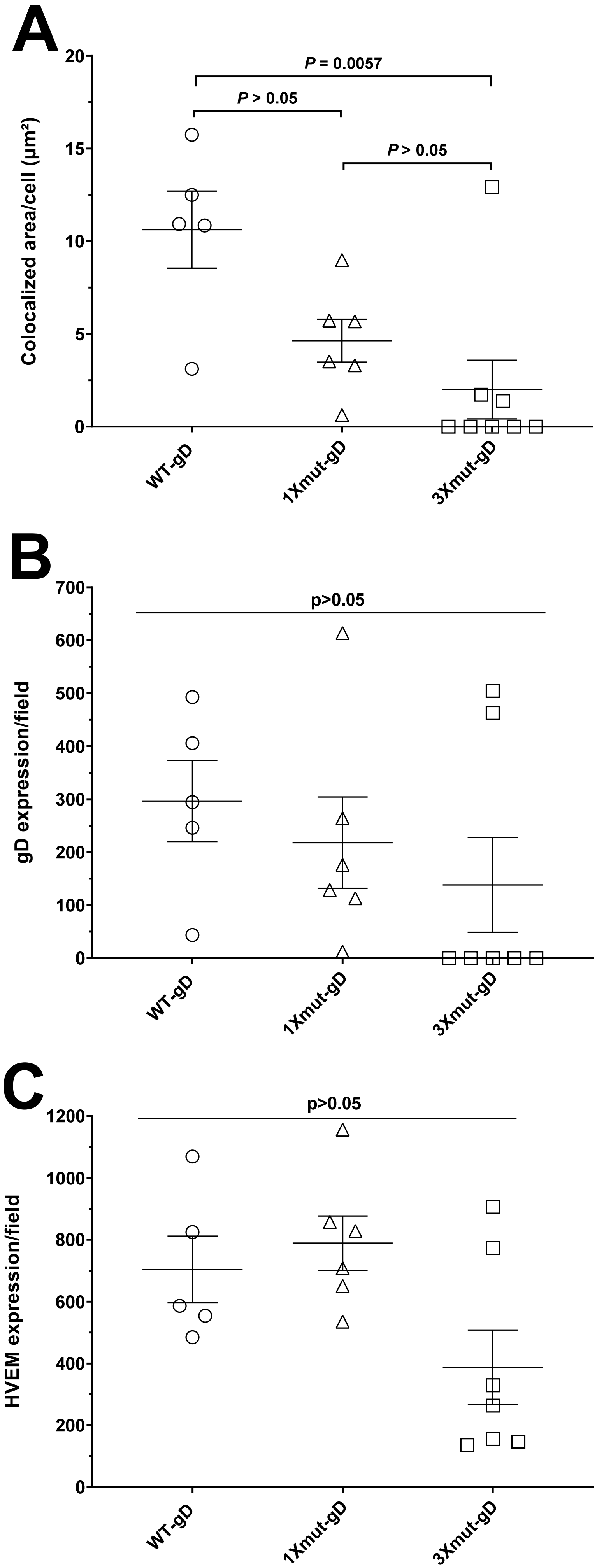
A
P = 0.0057
P > 0.05
P > 0.05
Colocalized area/cell (µm²)
20
15
10
5
0
WT-gD
1Xmut-gD
3Xmut-gD
B
p>0.05
gD expression/field
700
600
500
400
300
200
100
0
WT-gD
1Xmut-gD
3Xmut-gD
C
p>0.05
HVEM expression/field
1200
1000
800
600
400
200
0
WT-gD
1Xmut-gD
3Xmut-gD

Fig. S1

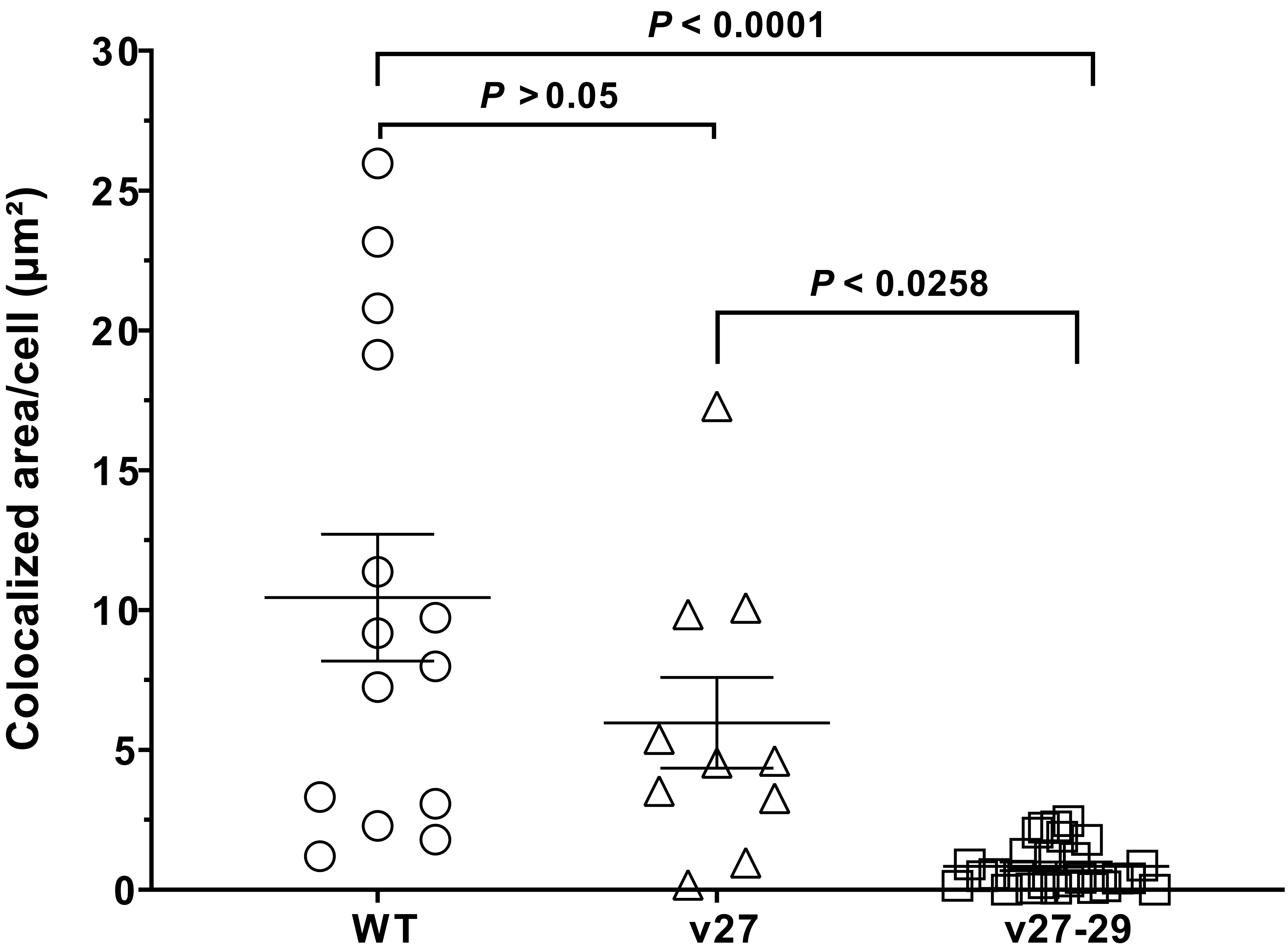

P < 0.0001
P >0.05
P < 0.0258
Colocalized area/cell (µm²)
30
25
20
15
10
5
0
WT
v27
v27-29

Fig. S2
