# Supplementary material for: Complete disruption of gD binding to HVEM alters virus replication and host receptor homeostasis
Source: PLoS Pathog. 2026 Sep 8;22(9):e1014566. doi: 10.1371/journal.ppat.1014566 (PMC13581205; doi:10.1371/journal.ppat.1014566)
Supplement: Supplementary file 3 [file ppat.1014566.s004.pdf]

3A	WT						v27						v27-29					
12	1740000	1650000	1730000	720000	2490000	1700000	4700000	3200000	2650000	2250000	3300000	2850000	4500000	5050000	1950000	7350000	2200000	2450000
24	34500000	20900000	8700000	8000000	7500000	9900000	10700000	11600000	9350000	3475000	4410000	2590000	1500000	5000000	2750000	1935000	1180000	1365000
48	34100000	33500000	47400000	14600000	23000000	17800000	41400000	50200000	25700000	20600000	22400000	16400000	48600000	69000000	45500000	24600000	22000000	20600000
3B																		
12	27300000	34800000	36800000	42400000	40800000	44200000	14800000	16700000	28500000	26500000	29400000	22700000	13400000	14600000	16900000	13800000	28800000	27800000
24	33300000	36350000	46150000	19000000	20700000	24200000	24300000	30150000	33100000	12300000	12700000	17300000	18350000	19900000	36350000	17800000	13200000	20500000
48	51700000	56300000	53800000	21800000	30400000	24600000	23500000	24300000	25500000	12600000	17400000	17800000	47300000	51200000	53900000	25600000	21600000	21400000
3C																		
12	3000						2000						2000					
24	30000	47000						30000	37000				20000	24000				
48		6000000	9200000				20000000	30000000	10200000					4000000				
3D																		
12	10000	20000					6000	20000					5000					
24	70000	67000					400000	110000	127000				150000	38000				
48	4000000	10200000					20000000	26000000	8200000				20000000	8000000	9400000			
3E																		
12	4000	20000					1000						10000					
24	20000	14000					20000	12000					20000					
48	2000000	1400000	720000				1400000	560000					1000000	860000				
3F																		
12	26000	20000					39000	10000					48000	70000				
24	90000	75000					120000	70000					130000	74000				
48	8000000	5600000					6000000	3400000					6000000	3800000				
3G																		
12	180000						130000						270000					
24	2400000	1120000					3200000	1310000					5200000	1580000				
48	60000000	14000000					40000000		11000000				40000000	18000000	15000000			
3H																		
12	370000						540000						410000					
24	1400000	780000					2800000	1250000					3800000	1370000				
48	10000000	10400000					20000000	16000000	10000000				40000000	16000000	12600000			

4A	WT			v27			v27-29		
12	1000			1000			1000		
24	60000	36000		19000			10000	9000	
48		3600000	2920000	1200000	1360000			1000000	1340000
4B									
12	12000	20000		11000	10000		5000	10000	
24	350000	174000		70000	86000		400000	140000	82000
48	80000000	16000000	6600000	40000000	48000000	32400000	3600000	1980000	

7A		WT			v27			v27-29		
24Hrs.		18677005	32826965	16151695	2243327	3585082	4687180	6367710	5982512	9084356
48Hrs.		346000000	347000000	286000000	15077300	19465860	14421140	16268091	19649556	23857783
7B										
24Hrs.		1189240	1487541	1456915	164396.2	204219.1	313197.9	445268.8	191745	378347.4
48Hrs.		41795905	61550206	34308888	1519636	1891033	1337990	1852246	1993421	2424997
7C										
24Hrs.		12628745	14142624	13437676	1537511	2037890	2055910	4511728	1592586	3222540
48Hrs.		453000000	479000000	438000000	17994464	22143232	12927563	17039020	21902375	29179322

8A	Mock		WT			v27			v27-29		
24Hrs.	0.89408	0.763527	1.364621	1.591046	1.260996	0.854878	1.011963	1.121017	1.290083	1.19969	1.257963
48Hrs.	1.192414	1	3.654057	4.56782	4.524874	2.174809	2.108662	1.698364	0.924496	0.65725	1.108702
8B											
24Hrs.	0.83789	0.700588	1.515351	1.580892	1.217803	0.763854	1.042908	1.089135	1.199789	1.13073	1.21579
48Hrs.	1.213085	1	0.734565	0.925991	0.985734	0.950281	0.739912	0.48774	0.519629	0.483236	0.476325
8C											
24Hrs.	0.824875	0.64003	1.365478	1.199845	1.058724	0.729504	0.904474	0.979728	1.08625	0.997304	1.129947
48Hrs.	0.96688	1	1.087136	1.193651	1.190008	0.763229	0.721272	0.482848	0.383732	0.406547	0.467661

9A	Mock		WT			v27			v27-29		
24Hrs.	2.18203	0.48914	2.27873	3.11019	1.25739	1.39676	0.66638	0.69702	2.75308	1.15317	2.46488
48Hrs.	1.24143	1	24.71	25.5806	12.3125	1.98286	2.80302	1.48087	4.11829	4.43887	4.74404
9B											
24Hrs.	0.44904		4.54395	15.4039	1.12509	0.35459		1.11978	2.01621	1.86581	1.28832
48Hrs.	1		78.1029	99.1851	67.2046	10.8738	4.86458	5.23505	7.12362	8.96484	9.29455
9C											
24Hrs.	0.86619	0.79158	1.14782	1.15853	0.97495	0.65706	0.44433	0.71392	0.73382	0.81894	0.47285
48Hrs.	0.67888	1	6.14199	6.26646	5.40133	2.26946	2.37667	2.283	1.46742	1.48666	1.51837
9D											
24Hrs.	1		0.32938	2.57858	0.86831	0.50341		0.80014	0.49683		1.30129
48Hrs.	1		8.50736	8.03664	2.12795	2.24364	1.78481	2.01587	2.56375	1.50866	2.37906

Fig.S1A				Fig.S1B				Fig.S1C		
**WT-Gd**	**Q27P-gD**	**Q27A-L28A-T29A-gD**		**WT-gD**	**Q27P-gD**	**Q27A-L28A-T29A-gD**		**WT-gD**	**Q27P-gD**	**Q27A-L28A-T29A-gD**
10.84634	8.984099	0		294.3201	613.533508	0		554.0743	535.466797	329.681366
12.4939	5.718864	0		405.734	264.289001	0		824.739	828.568237	136.082794
3.119572	3.308763	0		43.68042	113.209908	0		1069.542	857.521729	147.140808
10.93031	3.514068	1.72156		246.1562	128.494843	*2300.912842**		586.3118	1156.216309	156.018005
15.74035	5.677857	12.92623		492.6908	176.11998	462.75592		484.6218	707.95636	*4531.019531**
	0.619608	0			12.404984	0			650.710022	906.51947
		0				0				773.42572
		1.380254				504.563385				263.897736

Fig.S2		
WT	v27	v27-29
1.198165	4.607713	0.300753
	10.07831	0.867194
1.794337	4.536857	0.048613
3.074745	17.29196	1.78553
11.36695	0.971667	0.422168
7.250689	3.533667	0.003376
2.281276	3.270746	0.405132
9.174746	9.844859	0.000238
3.315619	5.394307	0.138277
9.726961	0.17532	2.227424
7.9925		1.900387
25.97087		0.48365
19.1307		1.284596
20.78809		2.044044
23.16574		0.035975
		1.14002
		0.910383
		0.121869
		2.455719
		2.266085
		0.505021
		1.178538
		0.237117
		0.398852
		0.06076
		0.564225
