# Supplementary material for: Complete disruption of gD binding to HVEM alters virus replication and host receptor homeostasis
Source: PLoS Pathog. 2026 Sep 8;22(9):e1014566. doi: 10.1371/journal.ppat.1014566 (PMC13581205; doi:10.1371/journal.ppat.1014566)
Supplement: Supplementary file 4 [file ppat.1014566.s006.doc]

**RE**: PPATHOGENS-D-26-01247

(Complete Disruption of gD Binding to HVEM Alters Virus Replication and Host Receptor Homeostasis)

Dear Drs. Jones, Kalejta, Bhaduri-McIntosh, and Malim:

Thank you very much for considering our revised manuscript. Below are our detailed responses to each reviewer's comments, indicating the changes we have made. Thank you for the opportunity to revise and resubmit our manuscript.

**Reviewer #1:**

HSV-1 gD binds the entry receptor HVEM; however, the specific contributions of individual amino acid residues in gD to this interaction remain incompletely understood. By generating targeted viral mutants, this study convincingly demonstrates that residues Q27 and Q27-T29 in gD are critical for HVEM binding, with the triple mutant disrupting this interaction more effectively than the Q27 single mutant and resulting in reduced receptor engagement as well as altered viral and host gene expression, particularly in neuronal cells.

**Issue 1**. Figure 1B: gD-HVEM colocalization appears to occur at or near the nuclear rim, which is unusual for these proteins. Please provide clarification or add in discussion. 1A: It is unclear whether the "1× mutant" and "3× mutant" correspond exactly to Q27 and Q27-29 mutants, respectively. If so, consistent terminology should be used throughout.

*RESPONSE*: We note that the signal may appear localized to the nuclear rim; however, it is actually present on the cell membrane. Confocal microscopy was performed, and 5–7 z-stacks were combined to improve visualization of the HVEM signal. Merging z-stacks can create the appearance of signal at the nuclear periphery, but this reflects membrane localization rather than true nuclear rim expression (line 200). To avoid any confusion in terminology, we have also replaced all instances of 1xmut and 3xmut with Q27P and Q27A-L28A-T29A, respectively.

**Issue 2**. Figure 2: The schematics are repetitive and could be consolidated into a single panel, potentially merged with Figure 1 to improve clarity.

*RESPONSE*: We wanted to highlight differences among the individual viral genomes; therefore, we included three separate schematic diagrams for the three viruses to avoid confusion.

**Issue 3**. Figure 3: Both logarithmic and linear scales are used interchangeably. A consistent approach (preferably logarithmic) is recommended; if not, please justify the rationale.

*RESPONSE*: The scales in both Fig. 3 and Fig. 4 have been converted to logarithmic scales.

**Issue 4**. Figure 4: Are the observed differences statistically significant? Please clarify.

*RESPONSE*: The *P* value is greater than 0.05 and is reported in the Results section (lines 178–181).

**Issue 5**. Figure 5: HVEM is supposed to be a transmembrane protein, but the authors observe strong nuclear localization (e.g., Fig. 5A, WT). This nuclear/nuclear rim localization remains puzzling and should be addressed.

*RESPONSE*: This has been explained in response to Issue 1.

**Issue 6**. Figure 6: The rationale for this experiment is not clearly explained, and its significance is difficult to interpret. Additionally, one might expect differences between the Q27 and Q27-T29 mutants, please comment.

*RESPONSE*: The rationale has been explained in the Results section, lines 215–218.

Thank you very much for allowing us to revise and resubmit our manuscript. We trust that we have satisfactorily addressed these helpful reviewers’ comments and have now made all the changes requested. New text in the body of the manuscript is printed in “Blue Arial” rather than “Black Arial.” We hope that the manuscript will now be deemed acceptable for publication in PLOS Pathogens.

Best Regards,

Homayon
