# Supplementary material for: Complete disruption of gD binding to HVEM alters virus replication and host receptor homeostasis
Source: PLoS Pathog. 2026 Sep 8;22(9):e1014566. doi: 10.1371/journal.ppat.1014566 (PMC13581205; doi:10.1371/journal.ppat.1014566)
Supplement: Supplementary file 5 [file ppat.1014566.s007.doc]

**RE**: PPATHOGENS-D-26-01247

(Complete Disruption of gD Binding to HVEM Alters Virus Replication and Host Receptor Homeostasis)

Dear Drs. Kalejta, Bhaduri-McIntosh, and Malim:

Thank you very much for considering our revised manuscript. Below are our detailed responses to each reviewer's comments, indicating the changes we have made. Thank you for the opportunity to revise and resubmit our manuscript.

**Reviewer #1:**

HSV-1 gD binds the entry receptor HVEM; however, the specific contributions of individual amino acid residues in gD to this interaction remain incompletely understood. By generating targeted viral mutants, this study convincingly demonstrates that residues Q27 and Q27-T29 in gD are critical for HVEM binding, with the triple mutant disrupting this interaction more effectively than the Q27 single mutant and resulting in reduced receptor engagement as well as altered viral and host gene expression, particularly in neuronal cells.

**Issue 1**. Figure 1B: gD-HVEM colocalization appears to occur at or near the nuclear rim, which is unusual for these proteins. Please provide clarification or add in discussion. 1A: It is unclear whether the "1× mutant" and "3× mutant" correspond exactly to Q27 and Q27-29 mutants, respectively. If so, consistent terminology should be used throughout.

*RESPONSE*: We note that the signal may appear localized to the nuclear rim; however, it is actually present on the cell membrane. Given that the gD-HVEM interaction occurs at the membrane of infected cells, live and non-permeabilized cells were stained and then fixed to assess only surface protein expression. For analysis, 8–10 z-stacks per field were acquired, and maximum-intensity projections were generated to visualize representative expression patterns. Merging z-stacks can create the appearance of signal at the nuclear periphery, but this reflects membrane localization rather than true nuclear rim expression (line 190-193). To avoid any confusion in terminology, we have also replaced all instances of 1xmut and 3xmut with Q27P and Q27A-L28A-T29A, respectively.

**Issue 2**. Figure 2: The schematics are repetitive and could be consolidated into a single panel, potentially merged with Figure 1 to improve clarity.

*RESPONSE*: We wanted to highlight differences among the individual viral genomes; therefore, we included three separate schematic diagrams for the three viruses to avoid confusion.

**Issue 3**. Figure 3: Both logarithmic and linear scales are used interchangeably. A consistent approach (preferably logarithmic) is recommended; if not, please justify the rationale.

*RESPONSE*: The scales in both Fig. 3 and Fig. 4 have been converted to logarithmic scales.

**Issue 4**. Figure 4: Are the observed differences statistically significant? Please clarify.

*RESPONSE*: The *P* value is greater than 0.05 and is reported in the Results section (lines 173–177).

**Issue 5**. Figure 5: HVEM is supposed to be a transmembrane protein, but the authors observe strong nuclear localization (e.g., Fig. 5A, WT). This nuclear/nuclear rim localization remains puzzling and should be addressed.

*RESPONSE*: This has been explained in response to Issue 1.

**Issue 6**. Figure 6: The rationale for this experiment is not clearly explained, and its significance is difficult to interpret. Additionally, one might expect differences between the Q27 and Q27-T29 mutants, please comment.

*RESPONSE*: The rationale has been explained in the Results section, lines 214–216.

**Reviewer #2:**

Arya and coworkers performed a series of studies to investigate with greater precision the binding of herpes simplex virus type 1 (HSV1)-encoded glycoprotein D (gD) to host cell receptor HVEM with focus on the contributions of individual amino acids to HVEM binding. Following construction of two mutant forms of HSV1 McKrae strain gD designated Q27P and Q27A – L28A – T29A that disrupt gD binding to HVEM, it was found that gD amino acids Q27 – T29 were critical for HVEM binding with the triple mutant showing greater disruption of HVEM binding than the Q27P single mutant. To further map gD binding to HVEM and assess functional relevance, two recombinant viruses were constructed on a HSV1 McKrae strain background and designated v27 virus (a single gD amino acid mutation) and v27 – 29 virus (three gD mutations). A series of in vitro studies were performed comparing thev27 virus, the v27 – 29 virus, and wildtype HSV1[McKrae] as a control for a series of experimental endpoints using four different mammalian cell lines that included rabbit skin cells, HeLa cells, mouse embryonic fibroblasts, and a neuronal cell line Neuro-2A. In contrast to the other three cell lines, HVEM protein expression was markedly reduced in v27 – 29-infected Neuro2a cells with no gD co-localization whereas Neuro-2A cells infected with v27 virus showed less HVEM and gD co-localization although gD protein levels remained unaffected. Additional studies showed that Neuro2A cells infected with a single or triple Gd mutant showed significantly less expression of several HSV1 glycoproteins that included gB and gC as well as gD, host cell receptors, and various HVEM ligands. The authors conclude that amino acids Q27 – T29 rather than Q27 alone are essential to completely block gD binding to HVEM.

Review: This is a well written manuscript that summarizes the findings of a series of relatively straightforward in vitro studies that provide convincing outcomes. These studies represent a nice companion to recently published work by this research group investigating gD and HVEM binding using the two recombinant viruses v27 and v27 – v29 in a mouse model of HSV1 corneal infection and latency. Attention to a few minor issues might strengthen the manuscript, however.

**Issue 1**. The authors use the word “significantly” to describe results but without providing the necessary p values. See Line 134 and Line 160. Later in the manuscript, use of the word “significantly” is supported by p values (Lines 172 – 174). The authors might consider using the word “markedly” in place of “significantly” when p values are not provided.

*RESPONSE*: P-values have been added at lines 134, 160, and 220. “Significantly” has been replaced with “markedly” on line 173.

**Issue 2**. Figures 4A and 4B show a comparison of the replication of wildtype virus, v27 virus, and v27 – 29virus in Neuro2a cells. The authors state that at an moi= 1.0, the replication of v27 – 29 virus in Neuro2a cells was significantly less (p > 0.05) than that of v27 virus at 48 hours postinfection and further state that the replication of v27 and wildtype viruses was “13 times higher” than that of v27 – 29 [Line 180]. However, inspection of Figure 4B suggests the amounts of infectious virus at 12 and 24 hours postinfection are relatively the same. How do the authors explain the more pronounced difference at 48 hour postinfection when gD and HVEM binding are early events? Is this difference due to progeny virus infections of neighboring uninfected cells (moi = 1.0 PFU/cell). Would this difference be observed at an moi = 10? Secondly, the comparisons are being made on a logscale. Neuro2a cells infected with the v27 – 29 virus nonetheless produce high amounts of infectious virus approaching 1 X 10^6 PFU/ml. Would not the decrease expected to be much greater? While “statistically significant” and perhaps a marked trend in reduction, would the findings be biologically relevant in the clinical setting?

*RESPONSE*: We thank the reviewer for these thoughtful comments regarding the replication kinetics in Neuro2a cells. At an MOI of 1.0, v27-29 showed a modest but statistically significant reduction in titer compared with v27 and WT at 48 hpi, despite similar titers at 12 and 24 hpi. We interpret this as a late-phase replication defect rather than a primary entry defect. Although gD-HVEM binding is an early event, the v27-29 mutations are expected to affect downstream processes such as cell-to-cell spread, secondary infection of neighboring cells, and possibly late gene expression or assembly, which become more evident at later time points. The larger difference at 48 hpi is consistent with impaired spread rather than a block in initial entry. We agree that at higher MOI (e.g., MOI = 10), where spread is less limiting, the difference between v27-29 and WT/v27 would likely be reduced. We focused on MOI = 1 to model conditions where cell-to-cell spread contributes to the overall titer. This has been explained in the Results section, lines 177-179.

**Reviewer #3:**

Overall, the strength of this study demonstrated that agD binding-site mutation, especially a triple aa mutation in gD, abolishes gD-HVEM binding or co-localization at the cell membrane. Although the gD binding-site mutation did not affect virus replication in non-neuronal cells (RS, HeLa cells, L929, MEF cells), itdid interfere with virus replication in Neuro 2A cells. In addition, the gD mutation in v27 or V27-v29 leads to reduced transcription of viral glycoproteins gB, gC, and gD, host receptors (HVEM, Nectin-1, and 3OS-SHtranscription, and HVEM ligands (LIGHT, LTz, CA160,and BTLA) compared to those infected with WT viruses.

The major concerns with the manuscripts are that the effects of the gD mutation were assessed (quantified) mostly at the transcription level, not at the protein production level. The mechanisms by which the gD mutation affects viral replication, transcription of host receptors, and receptor ligands were not discussed in detail. There was a difference in HVME ligand transcription in response to V27 and V27-29 virus infection at 48h. The transcription of Ltα in V27 virus-infected cells was upregulated more than that in V27-29 virus-infected cells. Could this be the reason why the V27 viral titer was similar to the WT virus titer at 48hpi? The terms gD-HVEM binding and co-localization were used synonymously during testing. What are the different effects of gD-HVEM binding or co-localization on the cell membrane versus the binding within the cytoplasm?

**Issue 1**. In Fig. 1A, the 3xmut-gD appears colocalized with HVEM in the cytoplasm of transfected cells, not on the cell membrane. How do you differentiate gD-HVEM binding or co-localization on the cell membrane or inside the cytoplasm? Is the gD trafficking to the membrane interfered by gD mutation?

*RESPONSE*: Fig. 1A shows the gD-HVEM interaction measured by a colocalization assay. In Fig. 1B, the same proteins are examined for their subcellular localization. As described in the Materials and Methods section, live and non-permeabilized cells were stained first and then fixed, allowing assessment primarily of surface-associated protein signal. In addition, images were acquired as 5–8 z-stacks per field and then projected into a single maximum-intensity image to enhance visualization of the otherwise weak signal, including in the WT condition (line 190-193; 414-433). In the mutant viruses, the signal is markedly reduced, which is why projection was used for display. This may give the appearance of higher intensity, but it reflects signal integration across the stack rather than increased expression. Therefore, the colocalization and localization shown in Fig. 1B, 5, and 6 should be interpreted as membrane-associated signal.

**Issue 2**. The mutation in gD could be illustrated in an expanded subfigure with precise site mutations in Fig.2.

*RESPONSE*: Fig.2 has been edited with wild type and mutant gD.

**Issue 3**. In Fig. 5, there were still many green stains at 48hpi. How do you tell if those were viral GFP signals or HVEM staining in the infected cells?

*RESPONSE*: HVEM was labeled with an Alexa Fluor 555-conjugated antibody to avoid signal overlap with GFP. In Fig. 5, HVEM is shown in green only for colocalization analysis and visualization purposes (line 414).

**Issue 4**. In Fig. 6, there were significant differences in the transcription of gB, gC, and gD among WT, v27, andv27-29 infections in Neuro2A cells. Still, the final viral titer of the v27 infection was similar to that of the WT infection. Why is the final viral titer the same between WT and v27 virus infection in the neuro2A cells? Were translations of gB, gC, and gD affected at 48hpi between WT and V27 virus infection?

*RESPONSE*: We thank the reviewer for this insightful comment. Fig. 4 shows replication reduction for the mutant viruses at 0.1 MOI, and for v27-29 at 1MOI. However, in Fig. 7, the mutant viruses-infected cells showed significant reduction in the copy numbers of viral genes. A plausible explanation is that transcript levels and final infectious output do not have to change in parallel. Final titer reflects the cumulative outcome of the whole replication cycle, so a transient drop in gB/gC/gD transcripts may not lower endpoint yield if late-stage assembly and release remain intact. This has been explained in the Results section, lines 231-235.

**Issue 5**. In Fig. 7, HVEM, Nectin-1, and 3OS-SH transcription at 48hpi were significantly lower in V27- and V27-29-virus-infected cells compared to WT virus-infected cells. However, it did not affect the virus titer at 48hpiin neuro2A cells infected with v27, except in cells infected with v27-29. Which of those receptors is compensating for the entry for V27 replication?

*RESPONSE*: If HVEM, nectin-1, and 3OS-SH transcripts are all reduced in v27- and v27-29-infected cells, that does not automatically mean entry is blocked, because: The virus may already have entered by 48 hpi, so receptor transcription at that time may be a consequence of infection rather than the cause of entry. Entry may have occurred earlier through pre-existing receptor protein on the cell surface, not the mRNA measured at 48 hpi. A reduction in receptor transcripts may not reduce titer if enough receptor protein remains or if nectin-1-mediated entry is still sufficient (line 284-292).

**Issue 6**. In Fig. 8, transcription of BTLA, LIGHT, Ltα, and CD160 was lower in the mutant virus infection compared to that in the WT virus infection. However, they all increased compared to the uninfected cells. The difference between v27 and v27-29 virus infection is not significant in the transcription of LIGHT andCD160. BTLA gene transcription had a slight increase inv27-v29 virus infection compared to v27-infected cells; on the other hand, Lymphotoxin-α had an increase in v27 infection compared to v27-29 infection. Therefore, the ligand response varies between v27 and v27-29. These differences were not highlighted in the results and discussion

*RESPONSE*: This has been explained in the Discussion section, lines 336-340.

**Issue 7**. 1: In the materials and methods section, the description of co-expression of gD and HVEM in infected cells included a fixation step after antibody staining. Is this the same procedure used in co-transfection staining?

*RESPONSE*: This is correct. The staining procedure was similar in both Neuro2a and HEK293T cells. Cells were stained first and then fixed.

**Issue 8**. lines 305-307: only v27-29 virus infection in neuro2A cells had reduced viral titer at 48hpi. The statement should be revised. How do you explain thev27 virus infection with no significant viral titer change but with a significant gB, gC, and gD transcription reduction?

*RESPONSE*: This has been explained in response to Issue 4.

**Issue 9**. lines 309-311, "the graded reduction ----" needs to be revised?

*RESPONSE*: This has been explained in the Discussion section, lines 314-316.

**Issue 10**. line 326-329. BTLA, LIGHT, Ltα, and CD160 gene expression were all higher in infected cells than in mock-infected cells. They were lower in v27- and v27-29-infected cells than in WT virus-infected cells. This means there was no downregulation in those mutant virus-infected cells, but there was a lack of upregulation of HVEM ligand transcription. The discussion on those HVEM ligand expressions needs to be revised accordingly.

*RESPONSE*: This has been explained in the Discussion section, lines 331-335.

**Reviewer #4:**

Herpes simplex virus type 1 (HSV-1) glycoprotein D (gD) plays a major player of virus entry, in large part because it interacts with heparan, HVEM. Previous studies revealed that amino acid 27-29 completely blocked gD binding to HVEM. This was the rational for mutating amino acid 27 and a mutant virus aa 27-29. These mutants were in the McKrae strain of HSV-1, which is a highly virulent strain of HSV-1. These mutant viruses were used to compare viral replication, corneal disease, latency, reactivation form latency, and T cell exhaustion. This study revealed that the ability of gD to interact with HVEM is crucial for viral replication and reactivation from latency. In general, this is an interesting study because interfering gD-HVEM can be used to develop a vaccine or small molecules that blocks the binding of HVEM to gD. With that said, there are a few minor issues that need to be addressed.

*RESPONSE*: The above comments and the following issues are not related to this study. In the current study we did not look at viral replication, corneal disease, latency, reactivation form latency, and T cell exhaustion in ocularly infected mice. In this study we compared these viruses *in vitro*. All of the following 5 minor issues are about the in vivo study and not the current study.

Minor issues:

**Issue 1**. The sentence on lines 27-29 needs to be revised because as is it indicates both mutant viruses were infected the same mice. “Mice infected with both mutant viruses had significantly lower eye disease, latency, and T cell exhaustion levels than observed in WT virus-infected mice.”

**Issue 2**. In Figure 1, the authors compare virus shedding when the eyes were scarified versus no scarification of wt MsKrae, the mutant containing only when position 27 was mutated (v27) or all 3 amino acids (v27-29) were mutated. As expected, both mutants had higher virus shedding when the eyes were scarified prior to infection. I was wondering if wt HSV-1 (McKrea) shed significantly higher than if not scarified? It looks like it does and the authors should consider stating whether the differences in virus shedding was significant or not.

**Issue 3**. Does HSV-2 gD contain the same amino acids at position of 27-29 of gD?

**Issue 4**. With respect to Figure 2, the authors should briefly explain how they measured corneal scarring and angiogenesis.

**Issue 5**. Figure 3 suggests there is no significant difference in LAT expression in TG regardless of scarification of the eyes of mice prior to no scarification. Can the authors comment on this?

Thank you very much for allowing us to revise and resubmit our manuscript. We trust that we have satisfactorily addressed these helpful reviewers’ comments and have now made all the changes requested. New text in the body of the manuscript is printed in “Blue Arial” rather than “Black Arial.” We hope that the manuscript will now be deemed acceptable for publication in PLOS Pathogens.

Best Regards,

Homayon
