# Supplementary material for: Complete disruption of gD binding to HVEM alters virus replication and host receptor homeostasis
Source: PLoS Pathog. 2026 Sep 8;22(9):e1014566. doi: 10.1371/journal.ppat.1014566 (PMC13581205; doi:10.1371/journal.ppat.1014566)
Supplement: Peer review history

# Peer review: Decision Letter 0

**DOI:** 10.1371/journal.ppat.1014566.r001

**Authors:** Robert Kalejta, Clinton Jones

10 Jul 2026

PPATHOGENS-D-26-01247

Complete Disruption of gD Binding to HVEM Alters Virus Replication and Host Receptor Homeostasis

PLOS Pathogens

Dear Dr. Ghiasi

Thank you for submitting your manuscript to PLOS Pathogens. After careful consideration, we feel that it has merit but does not fully meet PLOS Pathogens's publication criteria as it currently stands. Therefore, we invite you to submit a revised version of the manuscript that addresses the points raised during the review process.  It is recommended that the Authors address all of the minor revisions made by the three reviewers.  Fixing these minor concerns will improve this manuscript.

Please submit your revised manuscript by Aug 09 2026 11:59PM. If you will need more time than this to complete your revisions, please reply to this message or contact the journal office at plospathogens@plos.org. When you're ready to submit your revision, log on to https://www.editorialmanager.com/ppathogens/ and select the 'Submissions Needing Revision' folder to locate your manuscript file.

Please include the following items when submitting your revised manuscript:

* A letter that responds to each point raised by the editor and reviewer(s). You should upload this letter as a separate file labeled 'Response to Reviewers'. This file does not need to include responses to any formatting updates and technical items listed in the 'Journal Requirements' section below.

* A marked-up copy of your manuscript that highlights changes made to the original version. You should upload this as a separate file labeled 'Revised Manuscript with Track Changes'.

* An unmarked version of your revised paper without tracked changes. You should upload this as a separate file labeled 'Manuscript'.

If you would like to make changes to your financial disclosure, competing interests statement, or data availability statement, please make these updates within the submission form at the time of resubmission. Guidelines for resubmitting your figure files are available below the reviewer comments at the end of this letter.

As the corresponding author, your ORCID iD is verified in the submission system and will appear in the published article. PLOS supports the use of ORCID, and we encourage all coauthors to register for an ORCID iD and use it as well. Please encourage your coauthors to verify their ORCID iD within the submission system before final acceptance, as unverified ORCID iDs will not appear in the published article. Only the individual author can complete the verification step; PLOS staff cannot verify ORCID iDs on behalf of authors.

We look forward to receiving your revised manuscript.

Kind regards,

Clinton Jones, PhD

Academic Editor

PLOS Pathogens

Robert Kalejta

Section Editor

PLOS Pathogens

Sumita Bhaduri-McIntosh

Editor-in-Chief

PLOS Pathogens

orcid.org/0000-0003-2946-9497

Michael Malim

Editor-in-Chief

PLOS Pathogens

orcid.org/0000-0002-7699-2064

**Journal Requirements:**

If the reviewer comments include a recommendation to cite specific previously published works, please review and evaluate these publications to determine whether they are relevant and should be cited. There is no requirement to cite these works unless the editor has indicated otherwise.

1) In the online submission form, you indicated that The original contributions presented in this study are included in the article material. Further inquiries can be directed to the corresponding author.. All PLOS journals now require all data underlying the findings described in their manuscript to be freely available to other researchers, either

1. In a public repository

2. Within the manuscript itself

3. Uploaded as supplementary information.

This policy applies to all data except where public deposition would breach compliance with the protocol approved by your research ethics board. If your data cannot be made publicly available for ethical or legal reasons (e.g., public availability would compromise patient privacy), please explain your reasons by return email and your exemption request will be escalated to the editor for approval. Your exemption request will be handled independently and will not hold up the peer review process, but will need to be resolved should your manuscript be accepted for publication. One of the Editorial team will then be in touch if there are any issues.

2) Please amend your detailed Financial Disclosure statement. This is published with the article. It must therefore be completed in full sentences and contain the exact wording you wish to be published.

1) State what role the funders took in the study. If the funders had no role in your study, please state: "The funders had no role in study design, data collection and analysis, decision to publish, or preparation of the manuscript."

2) If any authors received a salary from any of your funders, please state which authors and which funders..

If you did not receive any funding for this study, please simply state: u201cThe authors received no specific funding for this work.u201d

3) We have amended your Competing Interest statement to comply with journal style. We kindly ask that you double check the statement and let us know if anything is incorrect.

4) PLOS now requires that authors provide the original uncropped and unadjusted images underlying all blot or gel results reported in a submission’s figures or Supporting Information files. This policy and the journal’s other requirements for blot/gel reporting and figure preparation are described in detail at https://journals.plos.org/plosone/s/figures#loc-blot-and-gel-reporting-requirements and https://journals.plos.org/plosone/s/figures#loc-preparing-figures-from-image-files.

When you submit your revised manuscript, please ensure that your figures adhere fully to these guidelines and provide the original underlying images for all blot or gel data reported in your submission. See the following link for instructions on providing the original image data: https://journals.plos.org/plosone/s/figures#loc-original-images-for-blots-and-gels.

In your cover letter, please note whether your blot/gel image data are in Supporting Information or posted at a public data repository, provide the repository URL if relevant, and provide specific details as to which raw blot/gel images, if any, are not available. Email us at customercare@plos.org if you have any questions.

**Reviewers' Comments:**

Reviewer's Responses to Questions

**Part I - Summary**

Please use this section to discuss strengths/weaknesses of study, novelty/significance, general execution and scholarship.

Reviewer #1: HSV-1 gD binds the entry receptor HVEM; however, the specific contributions of individual amino acid residues in gD to this interaction remain incompletely understood. By generating targeted viral mutants, this study convincingly demonstrates that residues Q27 and Q27-T29 in gD are critical for HVEM binding, with the triple mutant disrupting this interaction more effectively than the Q27 single mutant and resulting in reduced receptor engagement as well as altered viral and host gene expression, particularly in neuronal cells.

**********

**Part II – Major Issues: Key Experiments Required for Acceptance**

Please use this section to detail the key new experiments or modifications of existing experiments that should be absolutely required to validate study conclusions.

Generally, there should be no more than 3 such required experiments or major modifications for a "Major Revision" recommendation. If more than 3 experiments are necessary to validate the study conclusions, then you are encouraged to recommend "Reject".

Reviewer #1: N/A

**********

**Part III – Minor Issues: Editorial and Data Presentation Modifications**

Please use this section for editorial suggestions as well as relatively minor modifications of existing data that would enhance clarity.

Reviewer #1: Figure 1B: gD-HVEM colocalization appears to occur at or near the nuclear rim, which is unusual for these proteins. Please provide clarification or add in discussion. 1A: It is unclear whether the "1× mutant" and "3× mutant" correspond exactly to Q27 and Q27-29 mutants, respectively. If so, consistent terminology should be used throughout.

Figure 2: The schematics are repetitive and could be consolidated into a single panel, potentially merged with Figure 1 to improve clarity.

Figure 3: Both logarithmic and linear scales are used interchangeably. A consistent approach (preferably logarithmic) is recommended; if not, please justify the rationale.

Figure 4: Are the observed differences statistically significant? Please clarify.

Figure 5: HVEM is supposed to be a transmembrane protein, but the authors observe strong nuclear localization (e.g., Fig. 5A, WT). This nuclear/nuclear rim localization remains puzzling and should be addressed.

Figure 6: The rationale for this experiment is not clearly explained, and its significance is difficult to interpret. Additionally, one might expect differences between the Q27 and Q27-T29 mutants, please comment.

**********

PLOS authors have the option to publish the peer review history of their article (what does this mean?). If published, this will include your full peer review and any attached files.

If you choose “no”, your identity will remain anonymous but your review may still be made public.

**Do you want your identity to be public for this peer review?**  For information about this choice, including consent withdrawal, please see our Privacy Policy.

Reviewer #1: No

[NOTE: If reviewer comments were submitted as an attachment file, they will be attached to this email and accessible via the submission site. Please log into your account, locate the manuscript record, and check for the action link "View Attachments". If this link does not appear, there are no attachment files.]

**Figure resubmission:**

While revising your submission, we strongly recommend that you use PLOS’s NAAS tool (https://ngplosjournals.pagemajik.ai/artanalysis) to test your figure files. NAAS can convert your figure files to the TIFF file type and meet basic requirements (such as print size, resolution), or provide you with a report on issues that do not meet our requirements and that NAAS cannot fix.

After uploading your figures to PLOS’s NAAS tool - https://ngplosjournals.pagemajik.ai/artanalysis, NAAS will process the files provided and display the results in the "Uploaded Files" section of the page as the processing is complete. If the uploaded figures meet our requirements (or NAAS is able to fix the files to meet our requirements), the figure will be marked as "fixed" above. If NAAS is unable to fix the files, a red "failed" label will appear above. When NAAS has confirmed that the figure files meet our requirements, please download the file via the download option, and include these NAAS processed figure files when submitting your revised manuscript.

**Reproducibility:**

To enhance the reproducibility of your results, we recommend that authors of applicable studies deposit laboratory protocols in protocols.io, where a protocol can be assigned its own identifier (DOI) such that it can be cited independently in the future. Additionally, PLOS ONE offers an option to publish peer-reviewed clinical study protocols. Read more information on sharing protocols at https://plos.org/protocols?utm_medium=editorial-email&utm_source=authorletters&utm_campaign=protocols

# Author comment: Author response to Decision Letter 1

**DOI:** 10.1371/journal.ppat.1014566.r002

17 Jul 2026

Attachment Submitted filename: Response (07-14-2026)PP.doc

# Editor report: Decision Letter 1

**DOI:** 10.1371/journal.ppat.1014566.r003

**Authors:** Robert Kalejta, Clinton Jones

4 Aug 2026

PPATHOGENS-D-26-01247R1

Complete Disruption of gD Binding to HVEM Alters Virus Replication and Host Receptor Homeostasis

PLOS Pathogens

Dear Dr. Ghiasi,

Thank you for submitting your manuscript to PLOS Pathogens. After careful consideration, we feel that it has merit but does not fully meet PLOS Pathogens's publication criteria as it currently stands. Therefore, we invite you to submit a revised version of the manuscript that addresses the points raised during the review process.

Please submit your revised manuscript by Sep 03 2026 11:59PM. If you will need more time than this to complete your revisions, please reply to this message or contact the journal office at plospathogens@plos.org. When you're ready to submit your revision, log on to https://www.editorialmanager.com/ppathogens/ and select the 'Submissions Needing Revision' folder to locate your manuscript file.

Please include the following items when submitting your revised manuscript:

* A letter that responds to each point raised by the editor and reviewer(s). You should upload this letter as a separate file labeled 'Response to Reviewers'. This file does not need to include responses to any formatting updates and technical items listed in the 'Journal Requirements' section below.

* A marked-up copy of your manuscript that highlights changes made to the original version. You should upload this as a separate file labeled 'Revised Manuscript with Track Changes'.

* An unmarked version of your revised paper without tracked changes. You should upload this as a separate file labeled 'Manuscript'.

If you would like to make changes to your financial disclosure, competing interests statement, or data availability statement, please make these updates within the submission form at the time of resubmission. Guidelines for resubmitting your figure files are available below the reviewer comments at the end of this letter.

As the corresponding author, your ORCID iD is verified in the submission system and will appear in the published article. PLOS supports the use of ORCID, and we encourage all coauthors to register for an ORCID iD and use it as well. Please encourage your coauthors to verify their ORCID iD within the submission system before final acceptance, as unverified ORCID iDs will not appear in the published article. Only the individual author can complete the verification step; PLOS staff cannot verify ORCID iDs on behalf of authors.

We look forward to receiving your revised manuscript.

Kind regards,

Robert F. Kalejta, Ph.D.

Section Editor

PLOS Pathogens

Robert Kalejta

Section Editor

PLOS Pathogens

Sumita Bhaduri-McIntosh

Editor-in-Chief

PLOS Pathogens

orcid.org/0000-0003-2946-9497

Michael Malim

Editor-in-Chief

PLOS Pathogens

orcid.org/0000-0002-7699-2064

**Additional Editor Comments (if provided):**

Dear Dr. Ghiasi,

I need to inform you of an issue with the review of this manuscript. Your original decision letter contained only a single review. However, four reviews were obtained for the original manuscript. The fact that you did not receive the other reviews appears to be a clerical error. Nevertheless, the original reviews need to be addressed.

I have found your response to the comments of original reviewer #1 to be satisfactory.

Below please find the other three reviews. No additional experiments appear to be required, but each reviewer does ask for clarifications and changes to the manuscript.

Please address all comments of reviewers #2, #3, and #4 in a revised manuscript that will be editorially reviewed before a decision is rendered.

I apologize for the confusion and delay, and hope that we can quickly provide you with a suitable resolution to this unfortunate situation.

Reviewer #2

Arya and coworkers performed a series of studies to investigate with greater precision the binding of herpes simplex virus type 1 (HSV1)-encoded glycoprotein D (gD) to host cell receptor HVEM with focus on the contributions of individual amino acids to HVEM binding. Following construction of two mutant forms of HSV1 McKrae strain gD designated Q27P and Q27A – L28A – T29A that disrupt gD binding to HVEM, it was found that gD amino acids Q27 – T29 were critical for HVEM binding with the triple mutant showing greater disruption of HVEM binding than the Q27P single mutant. To further map gD binding to HVEM and assess functional relevance, two recombinant viruses were constructed on a HSV1 McKrae strain background and designated v27 virus (a single gD amino acid mutation) and v27 – 29 virus (three gD mutations). A series of in vitro studies were performed comparing the v27 virus, the v27 – 29 virus, and wildtype HSV1 [McKrae] as a control for a series of experimental endpoints using four different mammalian cell lines that included rabbit skin cells, HeLa cells, mouse embryonic fibroblasts, and a neuronal cell line Neuro-2A. In contrast to the other three cell lines, HVEM protein expression was markedly reduced in v27 – 29-infected Neuro2a cells with no gD co-localization whereas Neuro-2A cells infected with v27 virus showed less HVEM and gD co-localization although gD protein levels remained unaffected. Additional studies showed that Neuro2A cells infected with a single or triple gD mutant showed significantly less expression of several HSV1 glycoproteins that included gB and gC as well as gD, host cell receptors, and various HVEM ligands. The authors conclude that amino acids Q27 – T29 rather than Q27 alone are essential to completely block gD binding to HVEM.

This is a well written manuscript that summarizes the findings of a series of relatively straightforward in vitro studies that provide convincing outcomes. These studies represent a nice companion to recently published work by this research group investigating gD and HVEM binding using the two recombinant viruses v27 and v27 – v29 in a mouse model of HSV1 corneal infection and latency. Attention to a few minor issues might strengthen the manuscript, however.

1. The authors use the word “significantly” to describe results but without providing the necessary p values. See Line 134 and Line 160. Later in the manuscript, use of the word “significantly” is supported by p values (Lines 172 – 174). The authors might consider using the word “markedly” in place of “significantly” when p values are not provided.

2. Figures 4A and 4B show a comparison of the replication of wildtype virus, v27 virus, and v27 – 29 virus in Neuro2a cells. The authors state that at an moi = 1.0, the replication of v27 – 29 virus in Neuro2a cells was significantly less (p > 0.05) than that of v27 virus at 48 hours postinfection and further state that the replication of v27 and wildtype viruses was “13 times higher” than that of v27 – 29 [Line 180]. However, inspection of Figure 4B suggests the amounts of infectious virus at 12 and 24 hours postinfection are relatively the same. How do the authors explain the more pronounced difference at 48 hour postinfection when gD and HVEM binding are early events? Is this difference due to progeny virus infections of neighboring uninfected cells (moi = 1.0 PFU/cell). Would this difference be observed at an moi = 10? Secondly, the comparisons are being made on a log scale. Neuro2a cells infected with the v27 – 29 virus nonetheless produce high amounts of infectious virus approaching 1 X 10^6 PFU/ml. Would not the decrease expected to be much greater? While “statistically significant” and perhaps a marked trend in reduction, would the findings be biologically relevant in the clinical setting?

Reviewer #3

Overall, the strength of this study demonstrated that a gD binding-site mutation, especially a triple aa mutation in gD, abolishes gD-HVEM binding or co-localization at the cell membrane. Although the gD binding-site mutation did not affect virus replication in non-neuronal cells (RS, HeLa cells, L929, MEF cells), it did interfere with virus replication in Neuro 2A cells. In addition, the gD mutation in v27 or V27-v29 leads to reduced transcription of viral glycoproteins gB, gC, and gD, host receptors (HVEM, Nectin-1, and 3OS-SH transcription, and HVEM ligands (LIGHT, LTz, CA160, and BTLA) compared to those infected with WT viruses.

The major concerns with the manuscripts are that the effects of the gD mutation were assessed (quantified) mostly at the transcription level, not at the protein production level. The mechanisms by which the gD mutation affects viral replication, transcription of host receptors, and receptor ligands were not discussed in detail. There was a difference in HVME ligand transcription in response to V27 and V27-29 virus infection at 48h. The transcription of Ltα in V27 virus-infected cells was upregulated more than that in V27-29 virus-infected cells. Could this be the reason why the V27 viral titer was similar to the WT virus titer at 48hpi? The terms gD-HVEM binding and co-localization were used synonymously during testing. What are the different effects of gD-HVEM binding or co-localization on the cell membrane versus the binding within the cytoplasm?

The following are specific issues to be addressed:

1. In Fig. 1A, the 3xmut-gD appears colocalized with HVEM in the cytoplasm of transfected cells, not on the cell membrane. How do you differentiate gD-HVEM binding or co-localization on the cell membrane or inside the cytoplasm? Is the gD trafficking to the membrane interfered by gD mutation?

2. The mutation in gD could be illustrated in an expanded subfigure with precise site mutations in Fig. 2.

3. In Fig. 5, there were still many green stains at 48hpi. How do you tell if those were viral GFP signals or HVEM staining in the infected cells?

4. In Fig. 6, there were significant differences in the transcription of gB, gC, and gD among WT, v27, and v27-29 infections in Neuro2A cells. Still, the final viral titer of the v27 infection was similar to that of the WT infection. Why is the final viral titer the same between WT and v27 virus infection in the neuro2A cells? Were translations of gB, gC, and gD affected at 48hpi between WT and V27 virus infection?

5. In Fig. 7, HVEM, Nectin-1, and 3OS-SH transcription at 48hpi were significantly lower in V27- and V27-29-virus-infected cells compared to WT virus-infected cells. However, it did not affect the virus titer at 48hpi in neuro2A cells infected with v27, except in cells infected with v27-29. Which of those receptors is compensating for the entry for V27 replication?

6. In Fig. 8, transcription of BTLA, LIGHT, Ltα, and CD160 was lower in the mutant virus infection compared to that in the WT virus infection. However, they all increased compared to the uninfected cells. The difference between v27 and v27-29 virus infection is not significant in the transcription of LIGHT and CD160. BTLA gene transcription had a slight increase in v27-v29 virus infection compared to v27-infected cells; on the other hand, Lymphotoxin-α had an increase in v27 infection compared to v27-29 infection. Therefore, the ligand response varies between v27 and v27-29. These differences were not highlighted in the results and discussion

Minor comments:

1: In the materials and methods section, the description of co-expression of gD and HVEM in infected cells included a fixation step after antibody staining. Is this the same procedure used in co-transfection staining?

2. lines 305-307: only v27-29 virus infection in neuro2A cells had reduced viral titer at 48hpi. The statement should be revised. How do you explain the v27 virus infection with no significant viral titer change but with a significant gB, gC, and gD transcription reduction?

3. lines 309-311, "the graded reduction ----" needs to be revised?

4. line 326-329. BTLA, LIGHT, Ltα, and CD160 gene expression were all higher in infected cells than in mock-infected cells. They were lower in v27- and v27-29-infected cells than in WT virus-infected cells. This means there was no downregulation in those mutant virus-infected cells, but there was a lack of upregulation of HVEM ligand transcription. The discussion on those HVEM ligand expressions needs to be revised accordingly.

Reviewer #4

Herpes simplex virus type 1 (HSV-1) glycoprotein D (gD) plays a major player of virus entry, in large part because it interacts with heparan, HVEM. Previous studies revealed that amino acid 27-29 completely blocked gD binding to HVEM. This was the rational for mutating amino acid 27 and a mutant virus aa 27-29. These mutants were in the McKrae strain of HSV-1, which is a highly virulent strain of HSV-1. These mutant viruses were used to compare viral replication, corneal disease, latency, reactivation form latency, and T cell exhaustion. This study revealed that the ability of gD to interact with HVEM is crucial for viral replication and reactivation from latency. In general, this is an interesting study because interfering gD-HVEM can be used to develop a vaccine or small molecules that blocks the binding of HVEM to gD. With that said, there are a few minor issues that need to be addressed.

Minor issues:

1. The sentence on lines 27-29 needs to be revised because as is it indicates both mutant viruses were infected the same mice. “Mice infected with both mutant viruses had significantly lower eye disease, latency, and T cell exhaustion levels than observed in WT virus-infected mice.”

2. In Figure 1, the authors compare virus shedding when the eyes were scarified versus no scarification of wt MsKrae, the mutant containing only when position 27 was mutated (v27) or all 3 amino acids (v27-29) were mutated. As expected, both mutants had higher virus shedding when the eyes were scarified prior to infection. I was wondering if wt HSV-1 (McKrea) shed significantly higher than if not scarified? It looks like it does and the authors should consider stating whether the differences in virus shedding was significant or not.

3. Does HSV-2 gD contain the same amino acids at position of 27-29 of gD?

4. With respect to Figure 2, the authors should briefly explain how they measured corneal scarring and angiogenesis.

5. Figure 3 suggests there is no significant difference in LAT expression in TG regardless of scarification of the eyes of mice prior to no scarification. Can the authors comment on this?

**Journal Requirements:**

If the reviewer comments include a recommendation to cite specific previously published works, please review and evaluate these publications to determine whether they are relevant and should be cited. There is no requirement to cite these works unless the editor has indicated otherwise.

1) Please amend your detailed Financial Disclosure statement. This is published with the article. It must therefore be completed in full sentences and contain the exact wording you wish to be published.

i) State what role the funders took in the study. If the funders had no role in your study, please state: "The funders had no role in study design, data collection and analysis, decision to publish, or preparation of the manuscript."

ii) If any authors received a salary from any of your funders, please state which authors and which funders..

If you did not receive any funding for this study, please simply state: u201cThe authors received no specific funding for this work.u201d

**Reviewers' Comments:**

[NOTE: If reviewer comments were submitted as an attachment file, they will be attached to this email and accessible via the submission site. Please log into your account, locate the manuscript record, and check for the action link "View Attachments". If this link does not appear, there are no attachment files.]

**Figure resubmission:**

While revising your submission, we strongly recommend that you use PLOS’s NAAS tool (https://ngplosjournals.pagemajik.ai/artanalysis) to test your figure files. NAAS can convert your figure files to the TIFF file type and meet basic requirements (such as print size, resolution), or provide you with a report on issues that do not meet our requirements and that NAAS cannot fix.

After uploading your figures to PLOS’s NAAS tool - https://ngplosjournals.pagemajik.ai/artanalysis, NAAS will process the files provided and display the results in the "Uploaded Files" section of the page as the processing is complete. If the uploaded figures meet our requirements (or NAAS is able to fix the files to meet our requirements), the figure will be marked as "fixed" above. If NAAS is unable to fix the files, a red "failed" label will appear above. When NAAS has confirmed that the figure files meet our requirements, please download the file via the download option, and include these NAAS processed figure files when submitting your revised manuscript.

**Reproducibility:**

To enhance the reproducibility of your results, we recommend that authors of applicable studies deposit laboratory protocols in protocols.io, where a protocol can be assigned its own identifier (DOI) such that it can be cited independently in the future. Additionally, PLOS ONE offers an option to publish peer-reviewed clinical study protocols. Read more information on sharing protocols at https://plos.org/protocols?utm_medium=editorial-email&utm_source=authorletters&utm_campaign=protocols

# Author comment: Author response to Decision Letter 2

**DOI:** 10.1371/journal.ppat.1014566.r004

6 Aug 2026

Attachment Submitted filename: Response (08-05-2026)PP.doc

# Peer review: Decision Letter 2

**DOI:** 10.1371/journal.ppat.1014566.r005

**Authors:** Robert Kalejta, Clinton Jones

20 Aug 2026

Dear Dr. Ghiasi,

We are pleased to inform that your manuscript, "Complete Disruption of gD Binding to HVEM Alters Virus Replication and Host Receptor Homeostasis", has been editorially accepted for publication at PLOS Pathogens.

Before your manuscript can be formally accepted and sent to production, you will need to complete our formatting changes, which you will receive by email within a week. Please note that your manuscript will not be scheduled for publication until you have made the required changes.

IMPORTANT NOTES

(1) Copyediting and Proofreading: The corresponding author will receive a typeset proof for review, to ensure errors have not been introduced during production. Please review the PDF proof of your manuscript carefully, as this is the last chance to correct any errors. Please note that major changes, or those which affect the scientific understanding of the work, will likely cause delays to the publication date of your manuscript.

(2) Appropriate Figure Files: Please remove all name and figure # text from your figure files. Please also take this time to check that your figures are of high resolution, which will improve the readbility of your figures and help expedite your manuscript's publication. Please note that figures must have been originally created at 300dpi or higher. Do not manually increase the resolution of your files. For instructions on how to properly obtain high quality images, please review our Figure Guidelines, with examples at: http://journals.plos.org/plospathogens/s/figures.

We encourage you to use PACE (http://pace.apexcovantage.com/), a figure-checking tool which helps authors ensure all figures meet PLOS requirements so that figures can publish in the highest quality. PACE is a digital diagnostic and conversion tool for figure files. It will provide information about any failed check(s) and, if able, will automatically convert the figure into an acceptable file that passes quality checks.

To use PACE, you must first register as a user. Then, login and navigate to the UPLOAD tab, where you will find detailed instructions on how to use the tool.

(3) Striking Image: Please upload a striking still image to accompany your article if one is available (you can include a new image or an existing one from within your manuscript). Should your paper be accepted, this image will be considered for our monthly issue image and may also appear on our website to feature your article. Please upload this as a separate file, selecting "striking image" as the file type upon upload. Please also include a separate "Other" file with a caption, including credits and any potential copyright information. Please do not include the caption in the main article file. If your image is from someone other than yourself, please ensure that the artist has read and agreed to the terms and conditions of the Creative Commons Attribution License at http://journals.plos.org/plospathogens/s/content-license. Please note that PLOS cannot publish copyrighted images.

(4) Press Release or Related Media: If you or your institution will be preparing press materials for this manuscript, please inform our press team in advance at plospathogens@plos.org as soon as possible. We ask that you contact us within one week to plan ahead of our fast Production schedule. If you need to know your paper's publication date for related media purposes, you must coordinate with our press team, and your manuscript will remain under a strict press embargo until the publication date and time.

(5) Update your Profile Information: Now that your manuscript has been provisionally accepted, please log into Editorial Manager and update your profile, if needed. Go to http://www.editorialmanager.com/ppathogens, log in, and click on the "Update My Information" link at the top of the page. Please update your user information to ensure an efficient production and billing process.

(6) LaTeX users only: Our staff will ask you to upload a TEX file in addition to the PDF before the paper can be sent to typesetting, so please carefully review our Latex Guidelines http://journals.plos.org/plospathogens/s/latex in the meantime.

Thank you again for supporting Open Access publishing; we are looking forward to publishing your work in PLOS Pathogens.

Best regards,

Clinton Jones, PhD

Academic Editor

PLOS Pathogens

Robert Kalejta

Section Editor

PLOS Pathogens

Dominique Soldati-Favre

Editor-in-Chief

PLOS Pathogens

***********************************************************

Reviewer's Responses to Questions

**Part I - Summary**

Please use this section to discuss strengths/weaknesses of study, novelty/significance, general execution and scholarship.<br/><br/>

Reviewer #1: (No Response)

Reviewer #2: The authors have responded to all concerns and questions raised during the initial review process.

Reviewer #4: This is an important manuscript that provides novel insight into contributions of individual amino acid residues in gD to binding of the entry receptor HVEM and to viral and host gene expression. Available information demonstrates that the manuscript has been significantly modified and improved by following suggestions of reviewers #1 through #4. In my opinion the revised manuscript is sufficiently improved for publication.

The results of this study are highly relevant as they potentially lead to novel therapeutic modalities that through targeting the gD Q27-T29 region, or downstream HVEM-dependent signaling, can selectively reduce HSV-1 reactivation without substantially compromising control of primary infection.

--------------------

**Part II – Major Issues: Key Experiments Required for Acceptance**

Please use this section to detail the key new experiments or modifications of existing experiments that should be absolutely required to validate study conclusions.<br/><br/>Generally, there should be no more than 3 such required experiments or major modifications for a "Major Revision" recommendation. If more than 3 experiments are necessary to validate the study conclusions, then you are encouraged to recommend "Reject".<br/><br/>

Reviewer #1: (No Response)

Reviewer #2: None

Reviewer #4: No major issues: In my opinion the revised manuscript is sufficiently improved for publication.

--------------------

**Part III – Minor Issues: Editorial and Data Presentation Modifications**

Please use this section for editorial suggestions as well as relatively minor modifications of existing data that would enhance clarity.<br/><br/>

Reviewer #1: (No Response)

Reviewer #2: None

Reviewer #4: No minor issues: In my opinion the revised manuscript is sufficiently improved for publication.

--------------------

PLOS authors have the option to publish the peer review history of their article (what does this mean?). If published, this will include your full peer review and any attached files.

If you choose “no”, your identity will remain anonymous but your review may still be made public.

**Do you want your identity to be public for this peer review?**  For information about this choice, including consent withdrawal, please see our Privacy Policy.

Reviewer #1: No

Reviewer #2: No

Reviewer #4: No

# Editor report: Acceptance letter

**DOI:** 10.1371/journal.ppat.1014566.r006

**Authors:** Robert Kalejta, Clinton Jones

Dear Dr. Ghiasi,

We are delighted to inform you that your manuscript, "Complete Disruption of gD Binding to HVEM Alters Virus Replication and Host Receptor Homeostasis," has been formally accepted for publication in PLOS Pathogens.

We have now passed your article onto the PLOS Production Department who will complete the rest of the pre-publication process. All authors will receive a confirmation email upon publication.

Thank you again for supporting open-access publishing; we are looking forward to publishing your work in PLOS Pathogens.

Best regards,

Dominique Soldati-Favre

Editor-in-Chief

PLOS Pathogens
